# Bayesian multi-cell type models for the analysis of complex immune cell populations with application to ovarian cancer

**DOI:** 10.1093/bib/bbag053

**Published:** 2026-02-10

**Authors:** Chase J Sakitis, Jose Laborde, Julia Wrobel, Alex C Soupir, Christelle M Colin-Leitzinger, Benjamin G Bitler, Mary K Townsend, Andrew B Lawson, Joellen M Schildkraut, Shelley S Tworoger, Kathryn L Terry, Lauren C Peres, Brooke L Fridley

**Affiliations:** Division of Health Services and Outcomes Research, Children’s Mercy, 2401 Gillham Rd, Kansas City, MO 64108, United States; Biostatistics and Bioinformatics Shared Resource, Moffitt Cancer Center, 12902 USF Magnolia Drive, Tampa, FL 33612, United States; Department of Biostatistics, Rollins School of Public Health, Emory University, 201 Dowman Drive, Atlanta, GA 30322, United States; Department of Biostatistics and Bioinformatics, Department of Genitourinary Oncology, Moffitt Cancer Center, 12902 USF Magnolia Drive, Tampa, FL 33612, United States; Department of Cancer Epidemiology, Moffitt Cancer Center, 12902 USF Magnolia Drive, Tampa, FL 33612, United States; Department of Obstetrics and Gynecology, The University of Colorado Anschutz Medical Campus, 13001 E 17th Pl, Aurora, CO 80045, United States; Division of Oncological Sciences and the Knight Cancer Institute, Oregon Health and Science University, 3181 SW Sam Jackson Park Rd, Portland, OR 97239, United States; Department of Public Health Sciences, Medical University of South Carolina, 171 Ashley Avenue, Charleston, SC 29425, United States; Department of Epidemiology, Rollins School of Public Health, Emory University, 201 Dowman Drive, Atlanta, GA 30322, United States; Division of Oncological Sciences and the Knight Cancer Institute, Oregon Health and Science University, 3181 SW Sam Jackson Park Rd, Portland, OR 97239, United States; Department of Obstetrics and Gynecology, Brigham and Women’s Hospital and Harvard Medical School, 75 Francis St, Boston, MA 02115, United States; Department of Epidemiology, Harvard T. H. Chan School of Public Health, 677 Huntington Ave, Boston, MA 02115, United States; Department of Cancer Epidemiology, Moffitt Cancer Center, 12902 USF Magnolia Drive, Tampa, FL 33612, United States; Division of Health Services and Outcomes Research, Children’s Mercy, 2401 Gillham Rd, Kansas City, MO 64108, United States; School of Medicine, University of Missouri-Kansas City, 5000 Holmes St, Kansas City, MO 64110, United States

**Keywords:** Bayesian, cancer, dependency, hierarchical, spatial single-cell protein imaging data, tumor immune microenvironment, ovarian cancer

## Abstract

To understand how the tumor immune microenvironment (TIME) impacts clinical outcomes and treatment response, researchers have been leveraging single-cell protein multiplex imaging techniques. These technologies measure multiple protein markers simultaneously within a tissue sample, providing a more complete assessment of the TIME. However, statistical challenges arise from the over-dispersed and zero-inflated nature of the data and from relationships among different immune cell populations. To address these challenges, we developed a Bayesian hierarchical method using a beta-binomial (BB) distribution to model the abundance of multiple immune cell types simultaneously while incorporating relationships and immune cell differentiation paths. We applied the model to data from three large studies of high-grade serous ovarian tumors (Nurses’ Health Study I/II: *N* = 321, African American Cancer Epidemiology Study: *N* = 92, University of Colorado Ovarian Cancer Study: *N* = 103). We examined associations between cancer stage, age at diagnosis, and debulking status and the abundance of immune cell populations. We compared the multi-cell type model to individual cell type analyses using a Bayesian BB model. The multi-cell type model detected more associations, when present, with narrower credible intervals. To support broader application, we developed an R package, BTIME, with a detailed tutorial. In conclusion, the Bayesian multi-cell type model is flexible in how relationships between cell types are incorporated and can be used for cancer studies that interrogate the TIME.

## Introduction

Understanding the tumor immune microenvironment (TIME) is critical for advancing cancer research and improving therapeutic outcomes. The TIME encompasses a complex network of immune cells, stromal components, signaling molecules, and extracellular matrix elements that interact with tumor cells. These interactions can either suppress or promote tumor progression, influencing responses to immunotherapy (IO) [1–4] and other treatment modalities. By examining the cellular features within the TIME, researchers can identify prognostic biomarkers, uncover mechanisms of immune cell infiltration, and develop more effective, personalized therapeutic strategies.

A commonly used technology for immune profiling of the TIME is imaging-based spatial proteomics or multiplex immunofluorescence (mIF) [5, 6]. mIF allows for the assessment of multiple markers simultaneously and can be applied to archival tissue specimens, including tissue microarrays (TMAs), or whole slides. However, applying standard statistical modeling approaches to the mIF-derived cell abundance data remains challenging. First, in some cases, many tissue samples show no positive cells for a given marker, leading to zero-inflated data. Second, cell counts for different cell populations show over-dispersion from standard count-based data distributions [e.g. binomial (B) distribution]. Third, the cell populations measured in an experiment are often related and/or sub-populations of larger cell populations (i.e. CD3 + CD8+, cytotoxic T-cells with CD3 + CD8 + CD69+ cells or recently activated cytotoxic T-cells are all subsets of T-cells). Finally, experiments are often completed in which subjects can have multiple tissue samples (i.e. multiple cores within a TMA).

In this research, we sought to overcome these challenges through the development of novel Bayesian hierarchical statistical model and corresponding R software package BTIME. The Bayesian modeling framework simultaneously assesses the relationships between an exposure/predictor and each immune cell type, while incorporating relationships among immune cell populations. The models also account for the zero-inflated or over-dispersed nature of the count data using a beta-binomial (BB) model and accounts for repeated tissue samples per subject with a random subject effect. The utility of these Bayesian models was assessed in three large epidemiologic studies, in which multiple markers (e.g. CD3, CD4, CD8, FOXP3) representing different immune cell populations [e.g. total T-cells (CD3^+^), cytotoxic T-cells (CD3^+^CD8^+^)] were measured in epithelial ovarian cancer (EOC) tumors using mIF. The ability to apply these new methods to study the TIME of various cancers will allow for improved characterization and understanding of the complex immune landscape.

## Materials and methods

### Simulation study

**Figure 1:**
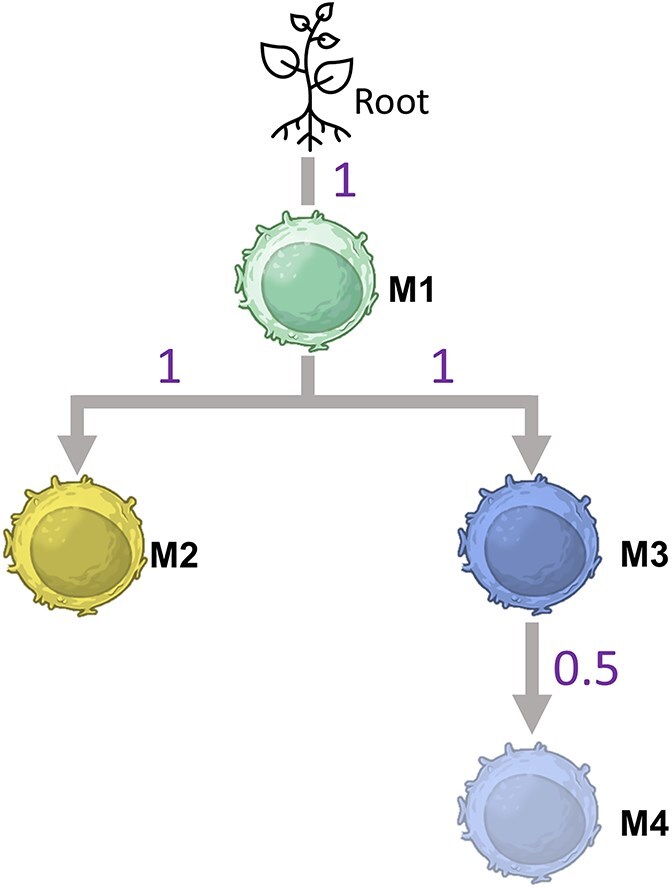
The hierarchical immune cell differentiation pathway showing the relationship between the cell populations for the simulated dataset.

We simulated multivariate BB count data using 50 samples split evenly between two groups defined by a binary predictor $X\in \left\{0,1\right\}$, representing a simplified, Low versus High, stage of cancer variable. For each sample, we generated counts for four cell types (M1, M2, M3, M4), each out of 500 total cells. The immune differentiation pathway for these four cell types is shown in Fig. 1. Baseline mean proportions in the control group were set to M1 = 0.25, M2 = 0.10, M3 = 0.01, M4 = 0.005 for the four cell types. Marker-specific dispersion was controlled using a common intra-class correlation of $\rho =0.05$, with Beta distribution parameters computed under the mean–dispersion reparameterization ${\alpha}_j={\mu}_j{\kappa}_j$, ${\beta}_j=\left(1-{\mu}_j\right){\kappa}_j$, and ${\kappa}_j=1/\rho -1$. In contrast to global group-level effects, we introduced local (per-sample) perturbations that modified only a subset of samples within each group (defined by the binary predictor). For a specified proportion of samples in each group, all four cell-type means were shifted by +25% for affected $X=1$ samples or − 25% for affected $X=0$ samples, resulting in locally higher or lower counts relative to baseline. We examined three perturbation levels: the null scenario or no affected samples (0%), a moderate level where 20% of each group was perturbed (5 samples per group), and a high level where 60% of each group was perturbed (15 samples per group).

Cell-type dependence was introduced using a Gaussian copula. A user-specified 4 × 4 target correlation matrix (shown in Equation 2.1) was first projected to the nearest positive-definite correlation matrix and could optionally be shrunk toward independence using ${R}_{\mathrm{cop}}=\left(1-s\right)I+s{R}_{\mathrm{target}}$. Latent Gaussian vectors $Z\sim{\mathcal{N}}_4\left(0,{R}_{\mathrm{cop}}\right)$ were then sampled and transformed to uniform variables $U=\Phi (Z)$, which were subsequently mapped to Beta-distributed probabilities using per-sample, per-marker shape parameters derived from the locally perturbed means. Conditional on these probabilities, marker counts were generated from B distributions, producing marginal BB variability. For each perturbation level (0%, 20%, and 60%), we ran 100 independent simulations, recording which samples were affected, the direction of the perturbation (‘up’ for $X=1$, ‘down’ for $X=0$), and the resulting raw counts and empirical rates.


(2.1) \begin{equation*} {R}_{\mathrm{target}}=\left[\begin{array}{@{}cc@{}}\begin{array}{@{}cc@{}}1.00& 0.80\\{}0.80& 1.00\end{array}&\!\!\!\!\!\!\!\! \begin{array}{@{}cc@{}}0.85& 0.70\\{}0.60& 0.50\end{array}\\{}\begin{array}{cc}0.85& 0.60\\{}0.70& 0.50\end{array}&\!\!\!\!\!\!\!\! \begin{array}{cc}1.00& 0.75\\{}0.75& 1.00\end{array}\end{array}\right] \end{equation*}

### Ovarian cancer studies

#### African American Cancer Epidemiology Study

African American Cancer Epidemiology Study (AACES) is a population-based case–control study of self-identified African American women with EOC residing in 11 geographic locations in the US and controls enrolled between 2010 and 2016 [7]. Centralized pathology review was completed to confirm diagnosis and histology. Formalin-fixed paraffin embedded (FFPE) tumor tissue blocks were collected, and TMAs were constructed, where multiple cores were sampled from each tumor. The parent protocol for the AACES study was approved by the Duke Health Institutional Review Board (IRB number Pro00022451).

#### University of Colorado Ovarian Cancer Study

FFPE tissue samples from 103 cancer patients with high-grade serous ovarian cancer (HGSOC) were collected at the University of Colorado Anschutz Medical Center (CU-AMC). Tissue cores were selected by a board-certified gynecologist to be included on the TMA [8, 9]. The protocol was reviewed and approved by the Colorado Multiple IRB #17-7788. Data for this study was downloaded from the Bioconductor package *VectraPolarisData* [10].

#### Nurses’ Health Study I and II

The Nurses’ Health Study (NHSI)/II was initiated in 1976 and prospectively enrolled 121,700 female registered nurses from 11 US states aged 30–55 years [11, 12]. In 1989, the NHSII was initiated and prospectively enrolled 116,429 female registered nurses from 14 US states aged 25–42 years [13]. For each individual with confirmed ovarian cancer that had a debulking surgery and a corresponding pathology report, FFPE tissue blocks were requested. A gynecologic pathologist selected regions with high-tumor content to be included in the TMAs [14]. The NHSI and NHSII protocols were approved by the IRBs of the Brigham and Women’s Hospital, Harvard T.H. Chan School of Public Health, and those of participating registries as required. Hathaway *et al.* provides further details about the NHSI and NHSII cohorts [15].

**Table 1:** Summary of the markers of interest assayed in the ovarian cancer studies used in this research study.

Study	Markers	Cell phenotype	Mean proportions (SD)	% of samples with 0 positive cells
AACES	CD3	T-cell/tumor infiltrating lymphocytes	**0.0534** (0.1006)	16%
	CD3+FOXP3+	Regulatory T-cell	**0.0067** (0.0139)	36%
	CD3+CD8+	Cytotoxic T-cell	**0.0172** (0.0407)	33%
UCOCS	CD3	T-cell/tumor infiltrating lymphocytes	**0.0346** (0.0588)	1%
	CD68	Macrophages	**0.0378** (0.0402)	0%
	CD19	B-cell	**0.0028** (0.0086)	26%
	CD3+CD8+	Cytotoxic-T cell	**0.0265** (0.0424)	1%
NHSI	CD3	T-cell/tumor infiltrating lymphocytes	**0.0316** (0.0645)	13%
	CD3+CD8+	Cytotoxic T-cell	**0.0126** (0.0298)	32%
	CD3+CD8+CD69+	Recently activated cytotoxic T-cell	**0.0041** (0.0108)	51%
	CD3+CD4+	Helper T-cell	**0.0075** (0.0321)	50%
	CD3+CD4+CD69+	Recently activated helper T-cell	**0.0019** (0.0134)	65%
	CD3+CD4+FOXP3+	Regulatory T-cell	**0.0021** (0.0115)	78%
	CD3+CD4+FOXP3+CD69+	Recently activated regulatory T-cell	**0.0005** (0.0048)	88%
NHSII	CD3	T-cell/tumor infiltrating lymphocytes	**0.0388** (0.0566)	8%
	CD3+CD8+	Cytotoxic T-cell	**0.0129** (0.0241)	18%
	CD3+CD8+CD69+	Recently activated cytotoxic T-cell	**0.0058** (0.0116)	31%
	CD3+CD4+	Helper T-cell	**0.0124** (0.0325)	33%
	CD3+CD4+CD69+	Recently activated helper T-cell	**0.0038** (0.0119)	48%
	CD3+CD4+FOXP3+	Regulatory T-cell	**0.0033** (0.0086)	64%
	CD3+CD4+FOXP3+CD69+	Recently activated regulatory T-cell	**0.0007** (0.0026)	76%

The bold values in the fourth column indicate the average proportion for each cell type in each study.

**Table 2:** Summary of the individuals and high-grade serous tumors included in the analysis by study.

	AACES	UCOCS	NHSI	NHSII
Number of subjects	92	103	252	69
Number of samples/cores	258	103	713	182
Cores per subject (SD)	2.8 (0.97)	1 (0)	2.83 (1.02)	2.64 (0.73)
Age at diagnosis (SD)	62.3 (9.61)	59.9 (10.42)	66.6 (9.64)	53.6 (7)
Stage (%)				
Early (Stage 1 or 2)	19.8	10.7	17.7	29.1
Late (Stage 3 or 4)	80.2	89.3	82.3	70.9
Debulking status				
Suboptimal	67	15	12	4
Optimal	111	65	26	12
Unknown	80	23	214	53

The markers assayed in each study are provided in Table 1 and a summary of participant characteristics is shown in Table 2. For each of these studies, mIF was performed using the AKOYA Biosciences OPAL™ 7-Color Automation IHC Kit. Following staining, image collection was performed by Vectra®3 Automated Quantitative Pathology Imaging System (0.499 μm/pixel). InForm was used for spectral unmixing, and HALO was used for cell phenotype assignments.

### Study preparation

For NHSI and NHSII, the samples were combined for the analysis performed in this research. Analyses were restricted to the tumor compartment of the cores, and any core with a tumor compartment <10 total cells was excluded. All analyses were also restricted to high-grade serous histotype (HGSOC), the most common type of EOC. This left 92 subjects and 258 cores for AACES and 321 subjects and 895 cores for NHSI/II. For University of Colorado Ovarian Cancer Study (UCOCS), analysis was completed on the tissue samples (*N* = 103 subjects, *N* = 103 cores) similar to prior published results [9]. Information on the cell abundance proportions are shown in Table 1. We evaluated the relationship of stage and age at diagnosis with the distributional assessment (i.e. immune cell abundance) using the Bayesian hierarchical model. For the analyses, stage was categorized as ‘Early’ (stages 1 and 2) and ‘Late’ (stage 3 and 4) and age at diagnosis was coded as a continuous variable in years. A subset of cases in AACES (70 subjects, 178 tissue cores) and UCOCS (80 subjects, 80 tissue cores) had information on debulking status, a critical treatment variable indicating whether residual tumor remains after surgical resection. Debulking status was modeled as a categorical variable with suboptimal representing ≥1 cm of residual tumor remaining after surgery and optimal representing <1 cm or no gross residual disease remaining after surgery. For NHSI/II, debulking status was only available in a limited number of cases (~17% between both studies) and used a different threshold (2 cm) for optimal debulking and thus not included in this study.

### Distributional model assessment for spatial proteomic imaging data

#### Discrete distributional models

The data obtained from the mIF images is the number of total cells measured within the tumor compartment of the sample and the number of cells positive for each marker or combination of markers denoting different immune cell types. Positivity is based on the staining intensity in the cell exceeding the marker-specific intensity threshold. Standard distributions for modeling this type of data would be either a B distribution or Poisson (P) distribution (in the case of rare events). However, these distributions do not directly account for the zero-inflated and/or over-dispersed nature of the cell count data. To account for over-dispersion, a BB or negative binomial (NB) distribution could be utilized to model the data. These two distributions do not directly account for the zero-inflation that can be observed in the case of rare cell populations. Distributions that model explicitly for zero-inflated counts include the zero-inflated binomial (ZIB) distribution and the zero-inflated Poisson (ZIP) distribution. Finally, to account for both the zero-inflated and over-dispersed nature that might be present in the data, the zero-inflated beta-binomial (ZIBB) and the zero-inflated negative binomial (ZINB) models could be used. These eight standard models were applied to the AACES, NHSI/II, and UCOCS mIF data, one cell type at a time, within a Bayesian framework for a preliminary assessment of distributional assumption for single-cell protein imaging data. To handle repeated measures involving multiple tissue cores per subject, a random subject effect was used to account for the dependence in multiple measurements taken from the same subject for the AACES and NHSI/II studies.

#### Assessment of discrete distributional model

The eight distributional models for cell type abundance measured using mIF were fit with stage as the predictor using the R packages ‘BRMS’ [16] and ‘RStan’ [17]. To assess model fit, we used leave-one-out cross-validation (LOOCV) and the widely applicable information criterion (WAIC) with Pareto-smoothed importance sampling (PSIS) as implemented in the ‘loo’ R package [18]. Priors were weakly informative distributions (i.e. default distributions in ‘BRMS’). Each chain was run for 14 000 iterations of the Markov chain Monte Carlo (MCMC) with the first 4000 iterations removed for burn-in and the last 10 000 iterations ($J$) were utilized for analysis. In total, four chains were run to monitor convergence, and the between (*B*) and within (*W*) chain variability was computed for each parameter. Using the between variance (*B*), the within variance (*W*), and the 10 000 sampling iterations ($J$), the Gelman–Rubin Convergence Diagnostic is utilized to estimate the Potential Scale Reduction Factor (PSRF), denoted as $\sqrt{\hat{R}}$, where $\sqrt{\hat{R}}=\sqrt{\left[\left(J-1/J\right)W+\left(1/J\right)B\right]/W}$. The PSRF is utilized as a convergence determinant with convergence achieved when $\sqrt{\hat{R}}<1.1$ [19] for each parameter of the model.

### Bayesian hierarchical beta-binomial model

From the preliminary distribution assessment analysis, the models accounting for over-dispersion (BB, NB, ZIBB, and ZINB) performed best. For the Bayesian hierarchical multi-cell type model, we chose a BB distributional model since it produced similar results to the other high performing models, is the simplest model to interpret and is applicable for cell types that do not exhibit zero inflation. Using the BB distributional model, the multi-cell type Bayesian model was extended to model all cell populations of interest simultaneously to allow borrowing of information between the different immune cell types. This comprehensive model also allows for explicit modeling of the biological relationship between cell types based on immune cell differentiation pathways.

The Bayesian multi-cell BB model is specified as follows. Let ${Y}_{igjk}$ represent the number of positive cells for cell type $i$ out of a total of ${N}_{gjk}$ cells measured in the core (sample) $k$ from subject $j$ in group $g$. Let the probability of positivity for cell type $i$ be represented by ${p}_{igj}$. The BB model is then specified as ${Y}_{igj k}\sim Bin\left({p}_{igj},{N}_{gjk}\right)$ with ${p}_{ig j}\sim Beta\left({\gamma}_i{\pi}_{ig},{\gamma}_i\left(1-{\pi}_{ig}\right)\right)$ where ${\gamma}_i>0$ with the prior distribution being a gamma distribution. To model the effect of a predictor on cell abundance ${Y}_{igjk}$ for subject $j$ in group $g$, we use the functional form $logit\left({\pi}_{ig}\right)={\beta}_0+{\beta}_1\left({X}_{gj}\right)+{S}_{1 igj}$ where ${S}_{1 igj}$ is a random subject effect to account for the repeated measures (i.e. multiple cores per subject). Gaussian prior distributions ${\beta}_0\sim MVN\left({v}_0,{T}_0\right)$ and ${\beta}_1\sim MVN\left({v}_1,{T}_1\right)$ are used to model the relationship between the $m$ immune cell types, where ${\beta}_0={\left({\beta}_{01},\dots, {\beta}_{0m}\right)}^T$ and ${\beta}_1={\left({\beta}_{11},\dots, {\beta}_{1m}\right)}^T$. Specified priors utilized for this work are discussed in the Supplemental material.

**Figure 2:**
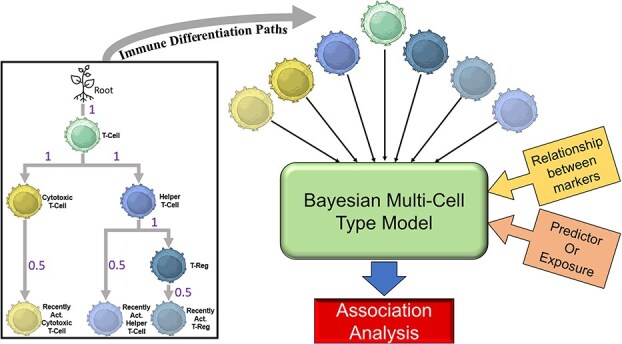
Illustration of the modeling framework for the Bayesian multi-cell type model using the protein markers from the NHSI/II ovarian cancer study. The hierarchical cell type tree or immune cell differentiation pathway (left) shows the relationship between the cell populations. The abundance of all cell populations is modeled simultaneously in the Bayesian multi-cell type model incorporating the relationship between cell populations.

Figure 2 exhibits a flowchart of the multi-cell modeling process using the immune cell types of interest from the NHSI/II. For the Bayesian model, we propose five different covariance prior models for modeling the relationships between the cell populations based on the markers and corresponding cell populations assessed: an unstructured covariance prior model (‘unstructured’), a covariance prior model based on distances in the immune cell differentiation path between cell types for correlation using an exponential spatial decay model (‘exponential decay’), and three tree-based models that utilizes the distances in the immune pathway (‘tree’, ‘scaled tree’, and ‘multi-level tree’ models). The black box on the left of Fig. 2 displays an example of a T-cell immune differentiation path and associated distances that can be used in different covariance prior models. In the NHSI/II study data, the distances were assigned as one for functional changes in the cell type and 0.5 for recent activation. However, these distances are flexible and can be set based on biological setting and cell populations being modeled (i.e. at the discretion of the researcher). Below we provide more information on approaches for modeling the relationship between the various cell populations in the study. For the unstructured model, the covariances ${\mathrm{T}}_0$ and ${\mathrm{T}}_1$ will follow inverse Wishart distributions. The best use case for the unstructured model is when the relationships between the cell types are unknown and the user wants to estimate relationships without imposing any subjective constraints.

#### Exponential decay covariance structure

The exponential decay model uses an exponential decay spatial relationship based on the distances between cell types via the cell differentiation pathway of the cell populations being assessed in the study. For this model, the covariance matrices are structured as ${\mathrm{T}}_0={\Sigma}_0\Delta{\Sigma}_0$, and ${\mathrm{T}}_1={\Sigma}_1\Delta{\Sigma}_1$, such that ${\Sigma}_0={\sigma}_{0m}{I}_m$, ${\Sigma}_1={\sigma}_{1m}{I}_m$ where ${\sigma}_{0m}=\sqrt{\sigma_{0m}^2}$, ${\sigma}_{1m}=\sqrt{\sigma_{1m}^2}$, ${\sigma}_{0m}^2$ and ${\sigma}_{1m}^2$ follow inverse gamma distributions, and $\Delta$ is the correlation matrix. The structure of the correlation matrix $\Delta$ is shown in Equation 2.2.


(2.2) \begin{equation*} \Delta =\left[\begin{array}{cc}\begin{array}{cc}1& {\rho}_{12}\\{}{\rho}_{21}& 1\end{array}& \begin{array}{cc}\cdots & {\rho}_{1m}\\{}\cdots & {\rho}_{2m}\end{array}\\{}\begin{array}{cc}\vdots & \vdots \\{}{\rho}_{m1}& {\rho}_{m2}\end{array}& \begin{array}{cc}\ddots & \kern0.75em \vdots \kern0.75em \\{}\cdots & 1\end{array}\end{array}\right] \end{equation*}

In this correlation matrix, ${\rho}_{st}=\exp \left(-{d}_{st}/\zeta \right)$ where ${d}_{st}$ is the immune differentiation distance between cell types $s$ and $t$, and $\zeta$ is the scale parameter, which follows a gamma distribution, that controls how quickly the correlation between the cell types decay. The distances between the cell types from the NHSI/II study are displayed in the black box of Fig. 2. For example, the distance between a T-cell (CD3) and a recently activated cytotoxic T-cell (CD3 + CD8 + CD69+) will be 1.5 since the distance values between the cell types add up to 1.5. The distances between the different steps of immune cell differentiation are determined by biological knowledge and at the researcher’s discretion. The ideal use of the exponential decay model is when the expected correlation between cell types decreases smoothly as their biological distance increases.

#### Tree covariance structure

The tree model will utilize the distances determined between cell populations as illustrated in Fig. 2 (including the root distance). These distances are then used to construct the covariance matrix $\mathrm{T}$. Instead of directly using the distances to construct the covariance matrix, this model utilizes lowest common ancestor (LCA) [20] to determine the variances and covariances between the cell populations. The structure of covariance matrix $\mathrm{T}$ is shown in Equation 2.3.


(2.3) \begin{equation*} \mathrm{T}=\left[\begin{array}{cc}\begin{array}{cc}{\tau}_{11}& {\tau}_{12}\\{}{\tau}_{21}& {\tau}_{22}\end{array}& \begin{array}{cc}\cdots & {\tau}_{1m}\\{}\cdots & {\tau}_{2m}\end{array}\\{}\begin{array}{cc}\vdots & \vdots \\{}{\tau}_{m1}& {\tau}_{m2}\end{array}& \begin{array}{cc}\ddots & \kern0.75em \vdots \kern0.75em \\{}\cdots & {\tau}_{mm}\end{array}\end{array}\right] \end{equation*}

In this covariance matrix, ${\tau}_{st}= LCA(s$,$t$) for cell types $s$ and $t$. The LCA method identifies the shared ancestor (node in the tree structure) between two cell types that is farthest from the root and uses that distance as the covariance. For example, between the cytotoxic T-cell (CD3 + CD8+ cell) and the recently activated cytotoxic T-cell (CD3 + CD8 + CD69+), they have two common nodes (ancestors): the T-cell (CD3+) and the cytotoxic T-cell. Then the distance to the root for both nodes is calculated to be 1 for the T-cell and 2 for the cytotoxic T-cell. The shared node farthest from the root is the cytotoxic T-cell, so that would be the covariance for ${\tau}_{st}$ between those cell types. With this tree structure, we can also set the covariance between cell types to be zero if the two cell types are not on the same branch. For example, the box on the left in Fig. 2 displays the differentiation path for the cell types in NHSI/II. As you move down the pathway away from the T-cell, the paths split down different branches where one branch starts with the cytotoxic T-cell (left side of the pathway) and the helper T-cell (right side of the pathway). With this ‘block’ design, any cell types that are on the opposite branches will have a zero covariance between them. This means that, for example, the covariance between an activated cytotoxic T-cell and a T-reg would be zero since they are on separate branches. The best case for using this covariance structure is when hierarchical lineage relationships dominates and the user wants a discrete tree-based relationship structure.

#### Scaled tree covariance structure

The scaled tree model utilizes the same covariance structure as the tree model, shown in Equation 2.3, but multiplied by a scalar. The equation for the covariance structure of this model is shown in Equation 2.4.


(2.4) \begin{equation*} \mathrm{T}=\lambda \ast \left[\begin{array}{cc}\begin{array}{cc}{\tau}_{11}& {\tau}_{12}\\{}{\tau}_{21}& {\tau}_{22}\end{array}& \begin{array}{cc}\cdots & {\tau}_{1m}\\{}\cdots & {\tau}_{2m}\end{array}\\{}\begin{array}{cc}\vdots & \vdots \\{}{\tau}_{m1}& {\tau}_{m2}\end{array}& \begin{array}{cc}\ddots & \kern0.75em \vdots \kern0.75em \\{}\cdots & {\tau}_{mm}\end{array}\end{array}\right] \end{equation*}

In this covariance matrix, ${\tau}_{st}= LCA(s$,$t$) for cell types $s$ and $t$ with the scalar $\lambda$ following a gamma distribution. By adding $\lambda$, it allows the data to appropriately scale the covariance matrix $\mathrm{T}$ without losing the structure of the matrix. The scaled tree model is ideally used when the user wants to use a tree-based relationship structure, but the overall strength of the relationship structure is uncertain, allowing a global scaling factor to adjust the magnitude according to the data.

#### Multi-level tree covariance structure

The multi-level tree model extends the methodology from the scaled tree model by separating the levels of the immune differentiation path displayed in Fig. 2. The equation for the covariance structure of the multi-level model is shown in Equation 2.5.


(2.5) \begin{align*} \mathrm{T}=&\ {\omega}_1\ast \left[\begin{array}{cc}\begin{array}{cc}{b}_{11}^{(1)}& {b}_{12}^{(1)}\\{}{b}_{21}^{(1)}& {b}_{22}^{(1)}\end{array}& \begin{array}{cc}\cdots & {b}_{1m}^{(1)}\\{}\cdots & {b}_{2m}^{(1)}\end{array}\\{}\begin{array}{cc}\vdots & \vdots \\{}{b}_{m1}^{(1)}& {b}_{m2}^{(1)}\end{array}& \begin{array}{cc}\ddots & \kern0.75em \vdots \kern0.75em \\{}\cdots & {b}_{mm}^{(1)}\end{array}\end{array}\right]+\cdots \nonumber \\ &+{\omega}_l\ast \left[\begin{array}{cc}\begin{array}{cc}{b}_{11}^{(l)}& {b}_{12}^{(l)}\\{}{b}_{21}^{(l)}& {b}_{22}^{(l)}\end{array}& \begin{array}{cc}\cdots & {b}_{1m}^{(l)}\\{}\cdots & {b}_{2m}^{(l)}\end{array}\\{}\begin{array}{cc}\vdots & \vdots \\{}{b}_{m1}^{(l)}& {b}_{m2}^{(l)}\end{array}& \begin{array}{cc}\ddots & \kern0.75em \vdots \kern0.75em \\{}\cdots & {b}_{mm}^{(l)}\end{array}\end{array}\right] \end{align*}

For this covariance structure, $l$ is the number of levels in the immune differentiation path, ${b}_{st}^{(l)}$ is either 0 or 1, and ${\omega}_l$ follows a gamma distribution. The ${b}_{st}^{(l)}$ binary value is based on whether cell types $s$ and $t$ cell types have a common node that is at or above the level $l$. For example, using the NHSI/II immune differentiation path shown in Fig. 2, we have 4 levels: T-cell in level 1, cytotoxic T-cell and helper T-cell in level 2, T-reg in level 3, and the recently activated cytotoxic T-cell, recently activated helper T-cell, and recently activated helper T-cell in level 4. If we wanted to get the $\mathrm{B}$ matrix for level 3, we would only place a 1 for the cell types $s$ and $t$ if both cell types contain the markers that make up a T-reg (CD3 + CD4 + FOXP3). For this level, the covariance between a T-cell and a T-reg would be zero since a T-cell only contains CD3+ and does not include CD4+ and FOXP3+. The T-reg would have a covariance of 1 with itself (its variance) and with the recently activated T-reg. In this third level, the variance for recently activated T-reg would also be one since it contains CD3 + CD4 + FOXP3. This model allows each level of the immune differentiation path to have its own weight, allowing more flexibility and aiding in determining which levels have higher effects on the covariance matrix. The multi-level tree model is most appropriate when different levels of the hierarchical tree structure contribute differently to the relationships between the cell types, enabling nuanced modeling of lineage effects.

#### Model assessment, convergence, and inference

For the simulation study, the Bayesian hierarchical multi-cell type models, along with the single-cell type analysis model, were fit using the R package ‘runjags’, which uses MCMC Gibbs sampling to estimate the posterior distribution [21], using similar priors for each model. Four chains were run with 40 000 iterations each, where the first 10 000 iterations were for the adaptation phase (initializing the model), the next 10 000 iterations were removed for burn-in, and the last 20 000 samples were utilized for analysis. Convergence was estimated using the PSRF (as previously described) along with the use of trace plots and was achieved when the $\sqrt{\hat{R}}$ of each parameter in the model is $<1.1$ [18] and the trace plots had low variability. To compare model performance between the 6 models (5 multi-cell models, 1 single-cell model), we use the *loo* R package for LOOCV as previously mentioned. To determine associations between the binary predictor and simulated abundance for each cell-type, we examined the ${\beta}_1$ parameter of the Bayesian models using 95% credible intervals (CI) using the posterior distribution.

For the experimental study with the 3 epidemiology studies, four chains were run with 202 000 iterations each, where the first 2000 iterations were the adaptation phase (initializing the model) and the next 100 000 were removed for burn-in, leaving 100 000 samples for analysis. Convergence was estimated using the PSRF (as previously described) along with the use of trace plots and was achieved when the $\sqrt{\hat{R}}$ of each parameter in the model is $<1.1$ [18] and the trace plots had low variability. To determine associations between predictor and immune cell infiltration for each phenotype, we only examined the ${\beta}_1$ parameter of the Bayesian models using 95% CI using the posterior distribution. For comparison between the multi-cell type and single-cell type model results, single-cell type models using the BB distribution were fit using similar priors and ‘runjags’ R package. We also included ‘block’ designs for the tree and scaled tree models for the NHSI/II analysis due to the datasets larger hierarchical structure in the immune differentiation pathway.

## Results

### Preliminary results

Assessment of the 8 discrete distributions for modeling cell abundance, involving the number of cells in the image and the number of cells positive for the different protein markers assayed in the studies, was completed using a Bayesian modeling framework with stage as the predictor (early/late). For the analysis of AACES and NHSI/II, random subject effects were included in the model to account for having multiple tissue cores per subject, with most subjects having 3 tissue cores (range 1–7 cores).

**Figure 3:**
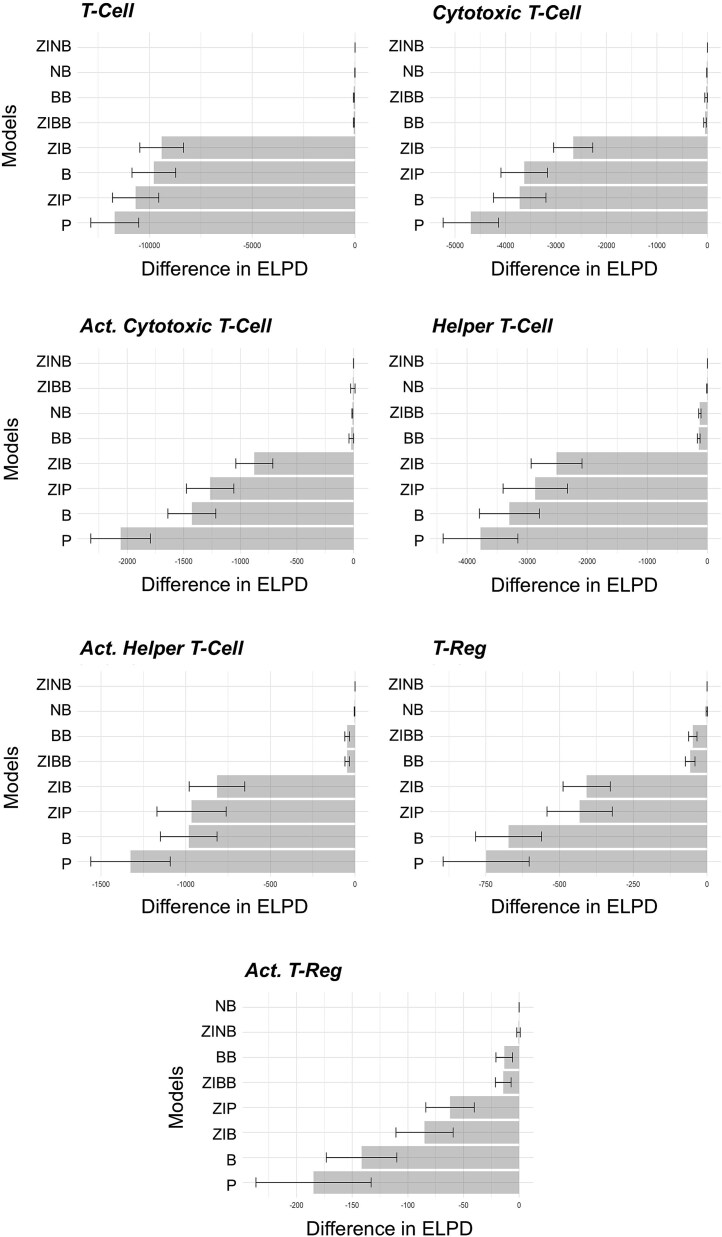
The difference in ELPD measurements from the best fitting model (top row in each plot) for the 8 discrete distributional models fit to the various immune markers assessed in the NHS/II study: B, P, BB, NB, ZIB, ZIP, ZINB, and ZIBB. Overall, the best fitting models were those where over-dispersion was explicitly modeled (ZIBB, ZINB, BB, and NB).

Figure 3 presents the differences in the expected log predictive density (ELPD) between the highest scoring model and all the other models for the NHSI/II. In model assessment, the model with the ELPD difference of zero indicates the preferred model [18]. In general, the eight standard models can be classified into 2 types: models where over-dispersion is explicitly modeled (BB, NB, ZIBB, ZINB) or models where over-dispersion is not explicitly modeled (B, P, ZIB, ZIP). In the NHSI/II, the over-dispersed models performed better in terms of model fit, as measured by smaller ELPD differences from the model with the highest ELPD (i.e. the best fitting model). The B and P distributional models were in general the worst performing models as they do not account for the excess variance or zero-inflation. A similar finding was observed in the analysis of the AACES and UCOCS (Supplementary Figs. S1 and S2, respectively). Hence, over-dispersion was a larger determinant of best fitting models than zero inflation. Zero-inflated distributions only worked well in the setting of an over-dispersed distribution. These results suggest that over-dispersed models fit spatial proteomic imaging data. Despite the zero-inflated, over-dispersed models (ZINB and ZIBB) performing the best, we used a BB model to develop the Bayesian hierarchical multi-cell type model as it performed only slightly worse than those two models while offering simpler interpretability (i.e. choose the simpler model over the more complex models involving zero-inflated terms).

### Simulation study results

For the simulation study, we first analyzed whether each model captured an association between the cell populations and the binary predictor variable for each of the 100 simulations. Table 3 shows the proportion of simulations that each model captured an association for each cell type in each perturbation level (high, moderate, and no effect). From this table, we can see that our multi-cell type model performed the best for the high perturbation level (60%) for each cell-type (3 for the scaled tree model and 1 for the tree model) while single-cell type analysis model performed the worst for each cell type. For the moderate perturbation level (20%), our multi-cell type model again captured more associations compared to the single-cell type model with the exponential decay model, the tree model, and the multi-level tree model performing the best for one cell type each and all 3 having the same capture rate for one of the cell types. For the perturbation with no effect, none of the models captured any associations between the simulated cell populations and the binary predictor variable.

**Table 3:** Proportion of the 100 simulations indicated as significant for each model and cell-type (based on 95% CI).

Perturbation level	Model	Cell-type 1 (M1)	Cell-type 2 (M2)	Cell-type 3 (M3)	Cell-type 4 (M4)
60% (15 subjects per group)	Single-cell	0.80	0.47	0.10	0.04
	Unstructured	0.81	0.50	0.11	0.05
	Exp. decay	0.83	0.52	0.11	0.05
	Tree	0.84	0.53	0.10	0.06
	Scaled tree	0.85	0.52	0.13	0.07
	Multi-level tree	0.83	0.50	0.12	0.06
20% (5 subjects per group)	Single-cell	0.07	0.07	0.05	0.04
	Unstructured	0.11	0.08	0.04	0.04
	Exp. decay	0.11	0.09	0.08	0.06
	Tree	0.13	0.08	0.08	0.06
	Scaled tree	0.12	0.08	0.06	0.05
	Multi-level tree	0.12	0.08	0.08	0.07
0% (No subjects)	Single-cell	0	0	0	0
	Unstructured	0	0	0	0
	Exp. decay	0	0	0	0
	Tree	0	0	0	0
	Scaled tree	0	0	0	0
	Multi-level tree	0	0	0	0

To further compare our multi-cell type model and the single-cell type model, we also evaluated the ELPD estimates (highest estimate indicates best model) and the range of the CIs for the ${\beta}_1$ parameter for each cell type in each simulation with the results displayed in Table 4. For the high perturbation level, our multi-cell type model had at least one of the models have the most simulations with the highest ELPD (two for the scaled tree model, one for the exponential decay model, and one for the tree model) and the narrowest CI (two for the exponential decay model, one for the scaled tree model, and one for the tree model). Similar results are shown for the moderate perturbation level as our multi-cell type model had at least one model with the most simulations that had the highest ELPD (two for the scaled tree model, one for the exponential decay model, and one for the tree model) and the narrowest CIs (one for the unstructured model, one for the exponential decay model, one for the scaled tree model, and one tie with the scaled tree and exponential decay models). The ELPD results for the no effect perturbation level are the same as the other perturbation levels for which model had the most simulations with highest ELPD. However, when evaluating the narrowest CIs for the no effect perturbation level, for M1, the single-cell type model had the most simulations while the unstructured model had the most for two of the cell types and the scaled tree model had one. Overall, each of our multi-cell type models performed well (except the unstructured for the most part) compared to the single-cell type model when considering the results from both Table 3 and Table 4. A sensitivity analysis of different distances between the cell types is discussed in the supplementary material with results shown in Table S1.

**Table 4:** Summary of the ELPD and CI ranges for each model and cell-type for each perturbation level from the 100 simulations.

		# of sim. with highest ELPD				# of sim. with narrowest CI			
Perturbation level	Model	M1	M2	M3	M4	M1	M2	M3	M4
60% (15 subjects per group)	Single-cell	9	13	0	0	9	18	2	0
	Unstructured	5	3	22	5	12	12	24	33
	Exp. decay	25	15	53	15	26	17	40	25
	Tree	7	5	16	76	15	8	19	34
	Scaled tree	29	34	5	4	19	30	11	8
	Multi-level tree	25	30	4	0	19	15	4	0
20% (5 subjects per group)	Single-cell	8	13	0	0	12	21	5	0
	Unstructured	12	8	25	4	14	11	26	48
	Exp. decay	19	9	48	14	21	18	42	14
	Tree	8	9	17	79	13	11	12	28
	Scaled tree	30	34	8	3	21	29	13	10
	Multi-level tree	23	27	2	0	19	10	2	0
0% (No subjects)	Single-cell	9	11	1	0	21	13	6	0
	Unstructured	14	9	28	8	15	17	41	53
	Exp. decay	18	10	34	10	16	17	23	12
	Tree	14	6	18	80	15	11	10	27
	Scaled tree	24	35	15	2	17	26	20	8
	Multi-level tree	21	29	4	0	16	16	0	0

### Comparison of single-cell type analysis to multi-cell type analysis

The Bayesian single-cell type models were fit using the ‘runjags’ R package to provide a consistent comparison to the Bayesian multi-cell type models using the AACES, UCOCS, and NHSI/II data. Along with modeling the five different Bayesian multi-cell type models, for the NHSI/II analysis, we also used the block designs (discussed in previous section) for the tree and scaled tree models, totaling 7 multi-cell type Bayesian models. For modeling the NHSI/II data, we also adjusted for cohort (NHSI versus NHSII). Assessment of convergence of the Bayesian single-cell type model followed the same procedure as completed for the Bayesian multi-cell type model as previously outlined. For comparison of the models, we compared the estimate of the effect for the predictor/covariate in the model (e.g. ${\beta}_1$).

**Figure 4:**
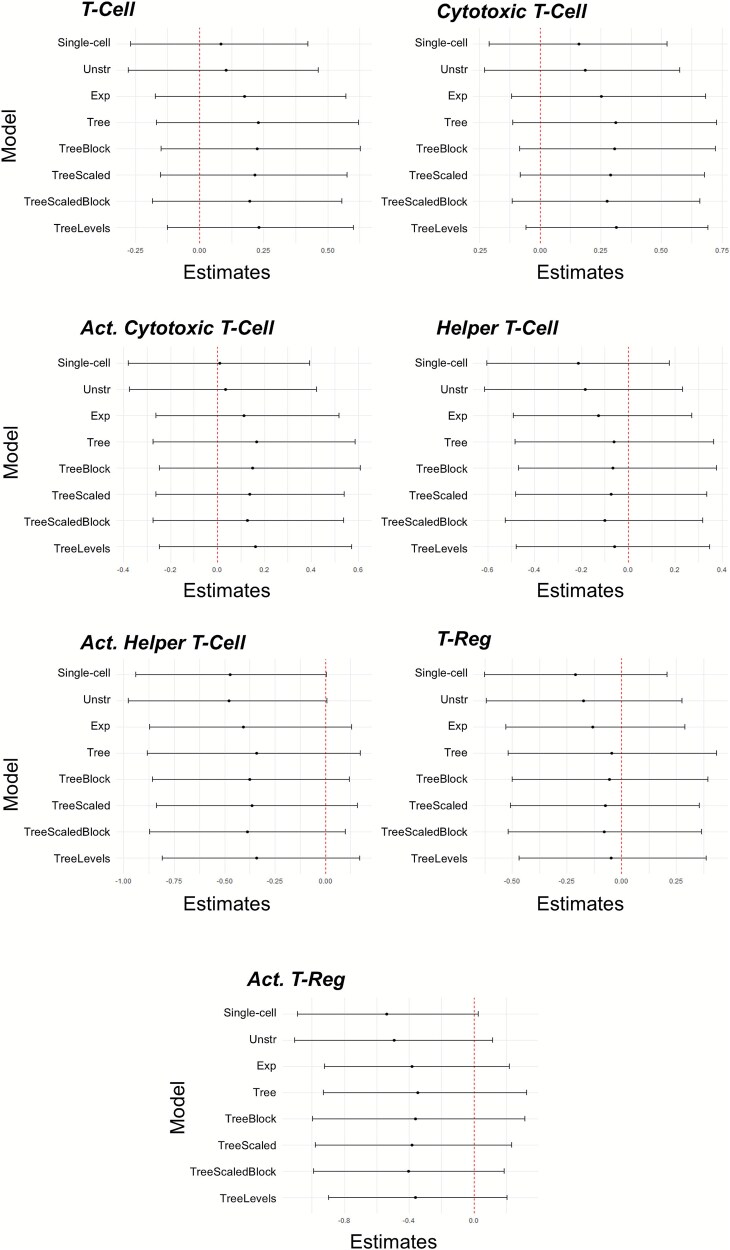
The 95% CIs for ${\beta}_1$ for each cell type from the analysis of the NHSI/II study for the single-cell type model (‘single-cell’) and the multi-cell type models assessing the association of stage of cancer (early versus late) with abundance. For the multi-cell type models, ‘Unstr’ is the unstructured covariance model, ‘Exp’ is the exponential decay covariance model, ‘tree’ is the tree covariance model, ‘TreeBlock’ is the tree covariance model with the block branch design, ‘TreeScaled’ is the scaled tree covariance model, ‘TreeScaledBlock’ is the scaled tree covariance model with the block branch design, and ‘TreeMultiLevel’ is the multi-level tree covariance model. The red dashed lines indicate the value of zero. Intervals that do not contain zero indicate a significant relationship between cancer stage and immune cell infiltration.

Results from the assessment of the impact of stage on cell abundance from the Bayesian multi-cell type and the single-cell type models are shown in Fig. 4. We observed no association of stage with immune cell abundance for any of the cell populations.

**Figure 5:**
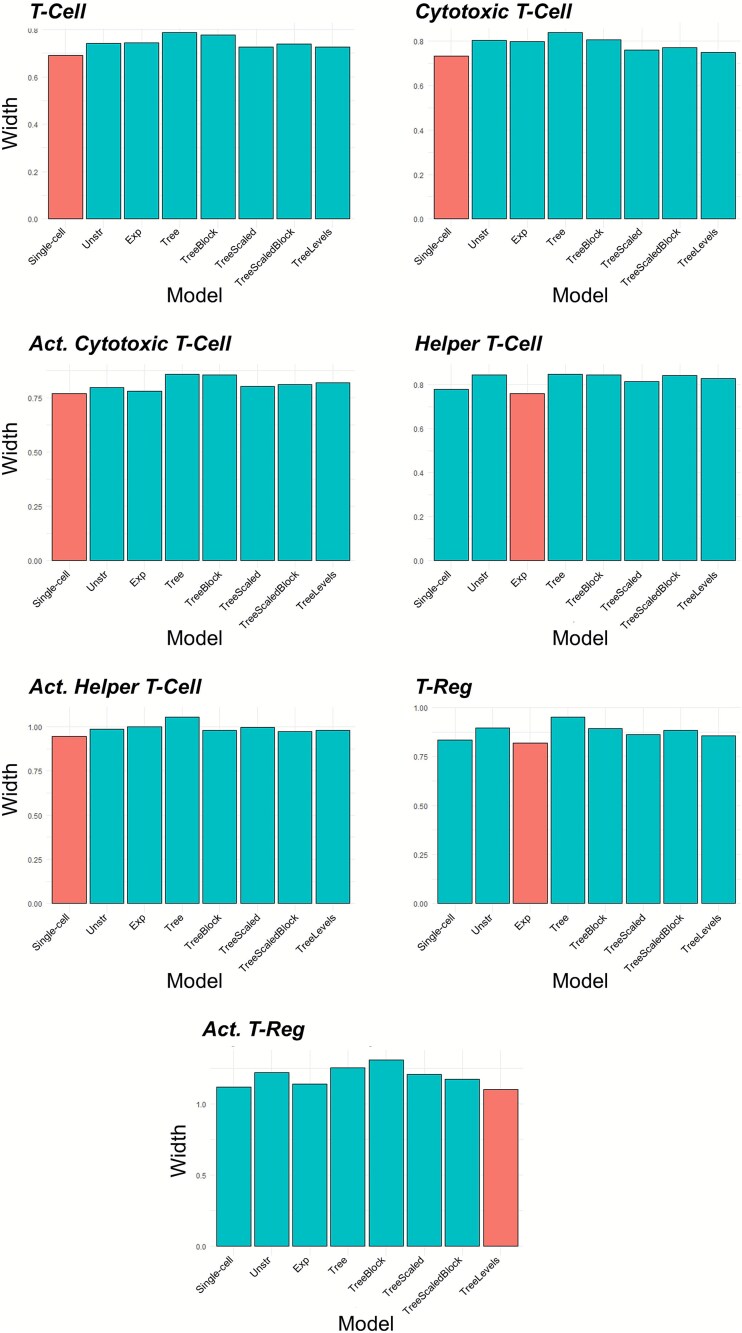
The width of the 95% CIs for ${\beta}_1$ for each cell type from the analysis of the NHSI/II study for the single-cell type model (‘single-cell’) and the multi-cell type models assessing the association of stage of cancer (early versus late) with abundance. For the multi-cell type models, ‘Unstr’ is the unstructured covariance model, ‘Exp’ is the exponential decay covariance model, ‘Tree’ is the tree covariance model, ‘TreeBlock’ is the tree covariance model with the block branch design, ‘TreeScaled’ is the scaled tree covariance model, ‘TreeScaledBlock’ is the scaled tree covariance model with the block branch design, and ‘TreeMultiLevel’ is the multi-level tree covariance model. The red bar indicates the model with the smallest width.

To further compare these models, the width of the CIs for ${\beta}_1$ for each cell type and model are plotted in Fig. 5. The results show that the single-cell type model had a slightly narrower CI for four of the cell types (T-cell, cytotoxic T-cell, recently activated cytotoxic T-cell, and recently activated helper T-cell) compared to the Bayesian multi-cell type models. Of the three cell types that the Bayesian multi-cell type model had narrower CIs, two of them (T-reg and recently activated T-reg) had extremely low abundance, demonstrating that borrowing strength from the other cell types is most helpful when modeling rarer cell populations.

**Figure 6:**
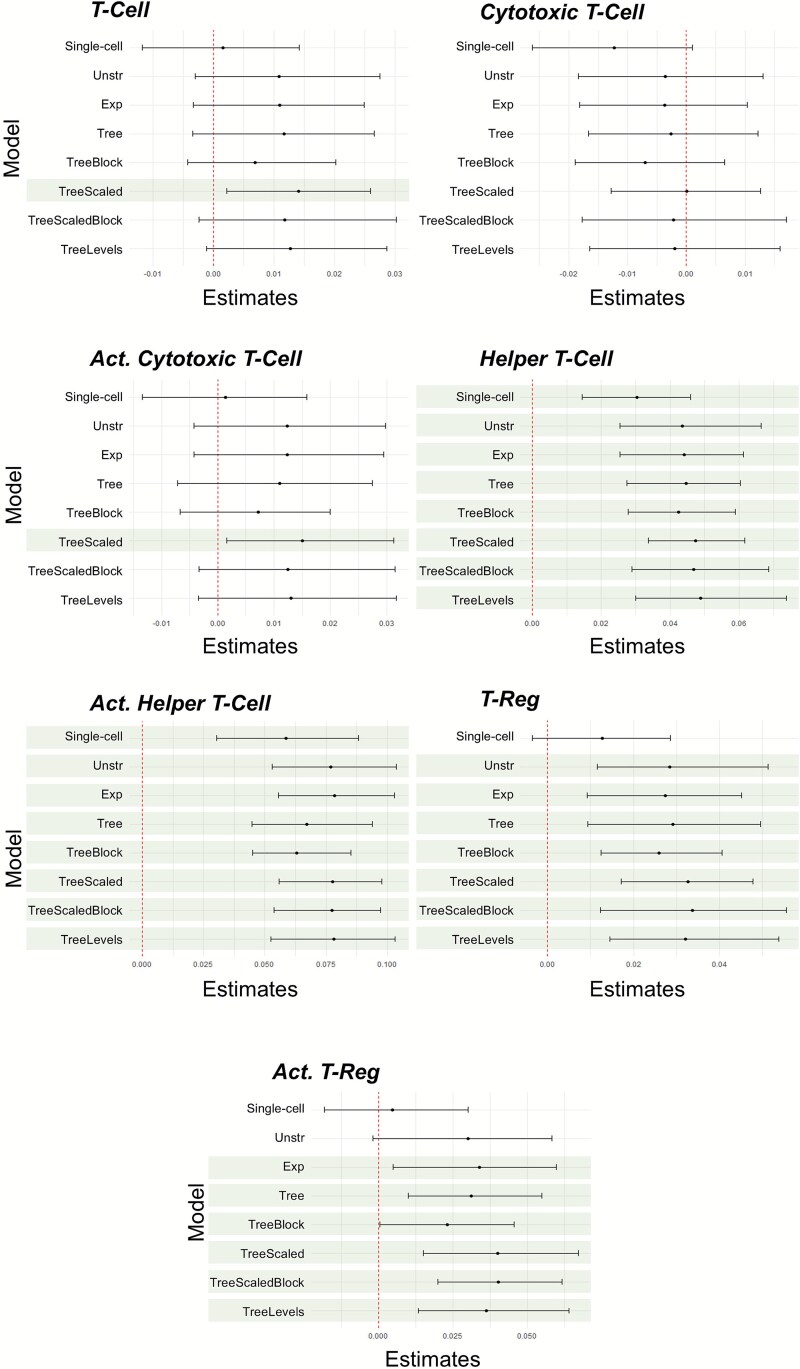
The 95% CIs for the ${\beta}_1$ estimates for each cell type from the NHSI/II analysis for the single-cell type analysis model (‘single-cell’) and the multi-cell type analysis models with age at diagnosis as the predictor. For the multi-cell type models, ‘Unstr’ is the unstructured model, ‘Exp’ is the exponential decay model, ‘tree’ is the tree model, ‘TreeBlock’ is the tree model with the block branch design, ‘TreeScaled’ is the scaled tree model, ‘TreeScaledBlock’ is the scaled tree model with the block branch design, and ‘TreeMultiLevel’ is the multi-level tree model. The red dashed lines for these plots indicate the x-intercept at zero. Intervals that do not contain zero indicate a significance between predictor (age) and immune cell infiltration. The intervals highlighted in green indicate significance between the immune cell population and cancer stage.

For the assessment of age at cancer diagnosis with immune cell infiltration, the 95% CIs for ${\beta}_1$ for each model of each cell type is presented in Fig. 6. We observed that all models detected an association of age at diagnosis with the abundance of helper T-cells and recently activated helper T-cells. For T-cells and recently activated cytotoxic T-cells, only the scaled tree model with the block design identified an association between cell abundance and age at diagnosis. For T-regs, all the multi-cell type models detected an association between age of cancer diagnosis and immune cell abundance, while the single-cell type model was unable to detect this association. For recently activated T-regs, the only models that did not detect an association with age of diagnosis were the single-cell type model and the multi-cell type model with an unstructured covariance relationship between cell types. When applied to the NHSI/II study data, the multi-cell type models outperform the single cell type model when capturing associations between immune cell populations and the predictor variable (age at diagnosis in this case) when there is an association present.

**Figure 7:**
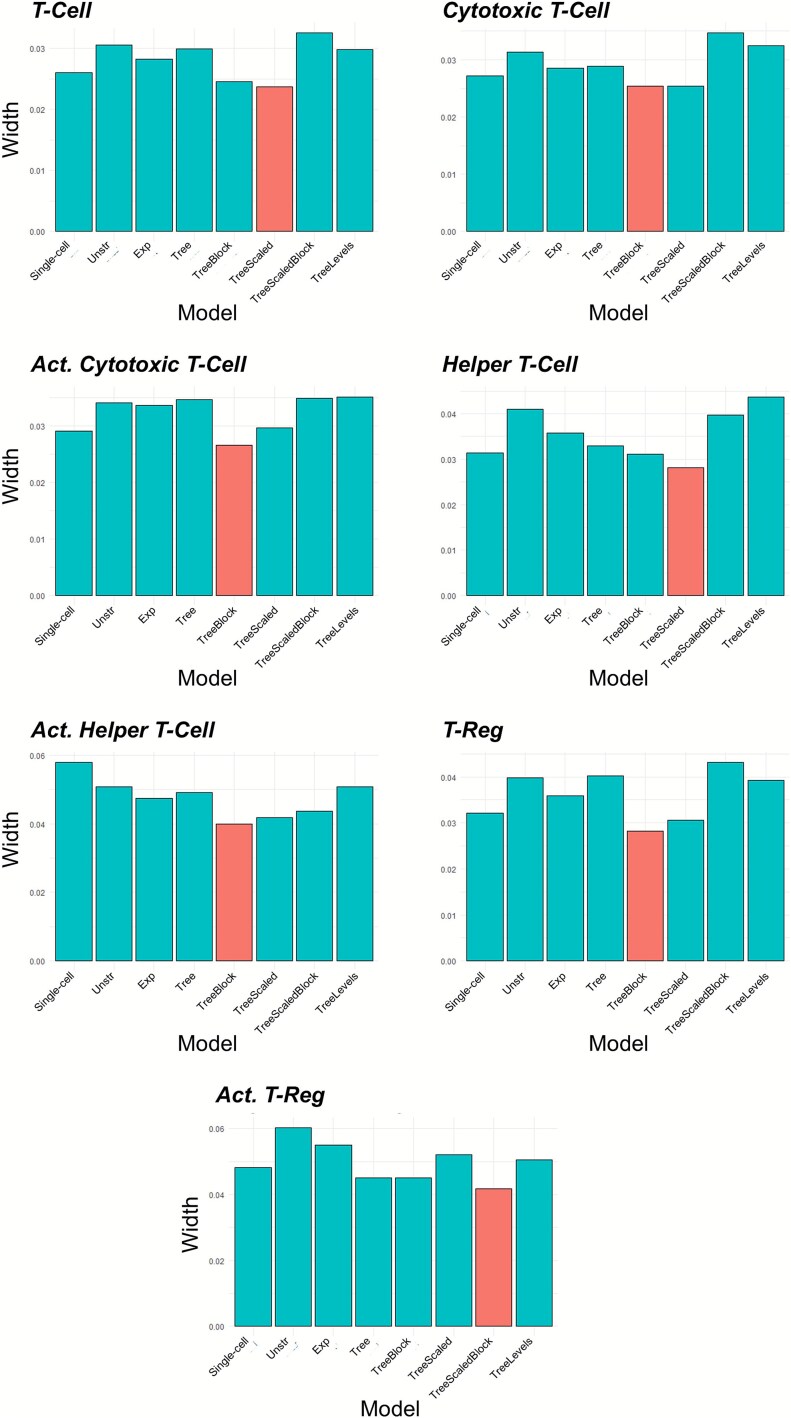
The width of the 95% CIs for ${\beta}_1$ for each cell type from the analysis of the NHSI/II study for the single-cell type model (‘Single-cell’) and the multi-cell type models assessing the association of age at cancer diagnosis with abundance. For the multi-cell type models, ‘Unstr’ is the unstructured covariance model, ‘Exp’ is the exponential decay covariance model, ‘Tree’ is the tree covariance model, ‘TreeBlock’ is the tree covariance model with the block branch design, ‘TreeScaled’ is the scaled tree covariance model, ‘TreeScaledBlock’ is the scaled tree covariance model with the block branch design, and ‘TreeMultiLevel’ is the multi-level tree covariance model. The red bar indicates the model with the smallest width.

The width of the ${\beta}_1$ CIs for the analyses of age at diagnosis are displayed in Fig. 7. The results show that one of the multi-cell type models across the different covariance structures had a smaller CI compared to the single-cell type model. This suggests that the multi-cell type models are able to borrow strength from the analysis of the other cell types through modeling the relationship between the cell populations.

Similar analyses evaluating stage, age at diagnosis, and debulking status as predictors were conducted in the AACES (Supplementary Figs. S6–S8) and UCOCS (Supplementary Figs. S9–S11). The analysis from these studies did not indicate any significant relationships for stage, age at diagnosis or debulking status with immune cell abundances. In comparing the width of the CIs between the single-cell type and multi-cell type models for AACES and UCOCS, the CIs were narrower for the multi-cell type models.

## Discussion

The goal of this research is to develop a Bayesian analysis framework for mIF data to facilitate the discovery of novel relationships between the TIME and clinical outcomes. Other Bayesian frameworks exist for analyzing the TIME, such as BayesTME [29], but differ in their objectives. BayesTME is a fully Bayesian, reference-free framework for spatial omics that integrates spot/cell deconvolution, phenotype discovery, and spatial differential expression within a single generative model, providing an end-to-end pipeline for inference and visualization. Its primary focus is on within-sample spatial deconvolution and latent phenotype discovery from raw spatial expression data, particularly in spatial transcriptomics. In contrast, our approach operates at the between-sample level, simultaneous analyzing multiple immune cell populations to determine their relationship with patient, tumor, or clinical features (i.e. IO response, stage of cancer, age at diagnosis).

This approach allows researchers to explicitly assess the relationship between the different cell populations based on known biology and immunology, such as immune cell differentiation pathways. Additionally, the modeling framework accounts for the over-dispersed and zero-inflated nature of immune cell abundances by using the BB model, which was one of the best-fitting models for a variety of immune cell populations over a range of abundances. Similar BB mixed-effects approaches have been developed for longitudinal discrete and bounded outcomes like patient-reported outcomes [30]. This method incorporates random effects to account for repeated measures and uses a frequentist estimation strategy, whereas our approach adopts a fully Bayesian framework, enabling posterior uncertainty quantification and flexible incorporation of clinical predictors. Furthermore, our model is tailored to multivariate immune cell abundances in the tumor microenvironment rather than single bounded outcomes, allowing simultaneous inference across multiple cell populations and their associations with patient-level covariates.

Our Bayesian model is flexible in how the relationships among different cell populations can be modeled. In this research, we examined five models for characterizing the relationship between cell types: unstructured relationship, exponential spatial decay, and three models that utilize a tree structure for modeling the relationship between cell populations. The exponential decay, the tree, the scaled tree, and the multi-level tree models were illustrated using the distance between cell types based on immune differentiation path for the available mIF data in NHSI/II, AACES, and UCOCS. The goal of these analyses was to assess the relationship of cancer stage, age at diagnosis, and debulking status (only for AACES and UCOCS) with the level of immune cell infiltration in the ovarian TIME. We found that age at diagnosis was associated with infiltration of recently activated cytotoxic T-cells and T-regs for all models considered (e.g. single-cell type model, multi-cell type model with different models for the relationship between cell populations). However, only the Bayesian multi-cell type models were able to detect an association with age at diagnosis and abundances of cytotoxic T-cells, helper T-cells, recently activated helper T-cells, and recently activated T-regs.

This relationship may reflect age-related changes in immune function, commonly referred to as immunosenescence, which can alter both innate and adaptive immune responses. Older individuals often exhibit increased levels of chronic low-grade inflammation (‘inflammaging’) [31], which could drive the recruitment or activation of specific immune subsets, such as macrophages or regulatory T cells, that promote a pro-tumor environment [32]. Conversely, if the associated cell types are cytotoxic or anti-tumor (e.g. CD8^+^ T cells or NK cells), this pattern might suggest a compensatory immune response attempting to counteract tumor progression in older patients [33]. These observations underscore the importance of considering age-related immune dynamics in ovarian cancer biology and may have implications for tailoring immunotherapeutic strategies based on age at cancer diagnosis.

When evaluating the width of the CIs for each cell type for both stage and age at diagnosis, in almost all cases, the multi-cell type model produced narrower intervals compared to the single-cell type model. No significant relationships were observed between age at diagnosis, cancer stage, or debulking status and immune cell levels for any of the models fit to the data from the AACES and UCOCS. However, it should be noted that some of the cell populations assessed in NHSI/II were not measured in AACES and UCOCS, particularly the activation marker (CD69); these studies also had a smaller sample size, reducing power.

This research presented a novel Bayesian multi-cell type model for modeling the cell populations form spatial protein imaging studies for studying the TIME. This framework is applicable to study the TIME of any tumor type, not just ovarian cancer. To facilitate the application of this modeling approach to other cancer studies conducted, we developed an R package BTIME to allow researchers to easily apply this modeling framework to their studies involving spatial protein imaging data. The Bayesian model is flexible and can be used for studies involving TMAs, or whole slide imaging data. Future work is on-going to apply this Bayesian model to studying the TIME of renal cell carcinoma, Ewing sarcoma, and hepatoblastoma with different features (i.e. IO response, recurrence/metastasis) and other immune cell populations. This model can help detect prognostic biomarkers related to TIME that could impact personalized treatment decisions like IO, which only shows therapeutic efficacy in a limited number of cancers and patients [22–28].

Lastly, we will be extending the model to allow the use of a zero-inflated BB model for settings where all the cell populations are extremely rare. The expansion of this modeling framework could aid in modeling diverse cell populations, connecting cell abundance to predictors/exposures.

**Key Points**

- Novel statistical approach: development of a Bayesian hierarchical model using a beta-binomial distribution to jointly analyze multiple immune cell types in the tumor immune microenvironment.
- Biological integration: the model incorporates known relationships and differentiation paths among immune cell populations, enhancing biological relevance and interpretability.
- Application to ovarian cancer studies: the method was applied to data from three large studies of high-grade serous ovarian tumors, demonstrating its utility across diverse cohorts.
- Improved analytical performance: compared to single-cell type models, the multi-cell type Bayesian approach detected more associations with clinical variables and produced narrower credible intervals.
- Software implementation: An R package, BTIME, was developed to facilitate broader use of the model in cancer research, complete with a detailed tutorial.

## Supplementary Material

BayesBB_Manuscript_Supplement_Final_bbag053

Supplementary_materials_bbag053

## Data Availability

The AACES data is available for use by contacting J. Schildkraut at Joellen.M.Schildkraut@Emory.edu. The University of Colorado study data can be found in the Bioconductor package VectraPolarisData. Because of participant confidentiality and privacy concerns, NHS/NHSII data are available upon reasonable written request. According to standard controlled access procedure, applications to use NHS/NHSII resources will be reviewed by an External Collaborators Committee for scientific aims, evaluation of the fit of the data for the proposed methodology, and verification that the proposed use meets the guidelines of the Ethics and Governance Framework and the consent that was provided by the participants. Investigators wishing to use NHS/NHSII data are asked to submit a description of the proposed project (https://www.nurseshealthstudy.org/researchers) or contact nhsaccess@channing.harvard.edu for details.

## Code availability

The R package for BTIME, used to complete the Bayesian hierarchical multi-cell model analyses for the AACES, NHSI/II, and Colorado ovarian cancer studies, is on CRAN or can be found at https://github.com/FridleyLab/BTIME.

## Notes

LCP reports grant funding from Bristol Myers Squibb, Janssen, and Karyopharm outside of the scope of this work. None of the other authors has a conflict of interest or financial disclosures to declare.
