# Supplementary material for: Bayesian multi-cell type models for the analysis of complex immune cell populations with application to ovarian cancer
Source: Brief Bioinform. 2026 Feb 10;27(1):bbag053. doi: 10.1093/bib/bbag053 (PMC12888822; doi:10.1093/bib/bbag053)

**SUPPLEMENTAL MATERIAL**

**Title**: Bayesian multi-cell type models for the analysis of complex immune cell populations with application to ovarian cancer

**Authors**: Chase J. Sakitis^1^, Jose Laborde^2^, Julia Wrobel^3^, Alex C. Soupir^4^, Christelle M. Colin-Leitzinger^5^, Benjamin G. Bitler^6^, Mary K. Townsend^7^, Andrew B. Lawson^8^, Joellen M. Schildkraut^9^, Shelley S. Tworoger^7^, Kathryn L. Terry^10,11^, Lauren C. Peres^5^, Brooke L. Fridley^1,12^

^1^Division of Health Services & Outcomes Research, Children’s Mercy, Kansas City, MO, USA

^2^Biostatistics and Bioinformatics Shared Resource, Moffitt Cancer Center, Tampa, FL, USA

^3^Department of Biostatistics, Rollins School of Public Health, Emory University, Atlanta, GA, USA

^4^Department of Biostatistics and Bioinformatics, Department of Genitourinary Oncology, Moffitt Cancer Center, Tampa, FL, USA

^5^Department of Cancer Epidemiology, Moffitt Cancer Center, Tampa, FL, USA

^6^Department of Obstetrics and Gynecology, The University of Colorado Anschutz Medical Campus, Aurora, CO, USA

^7^Division of Oncological Sciences and the Knight Cancer Institute, Oregon Health and Science University, Portland, OR, USA

^8^Department of Public Health Sciences, Medical University of South Carolina, Charleston, SC, USA

^9^Department of Epidemiology, Rollins School of Public Health, Emory University, Atlanta, GA, USA

^10^Department of Obstetrics and Gynecology, Brigham and Women’s Hospital and Harvard Medical School, Boston, MA, USA

^11^Department of Epidemiology, Harvard T. H. Chan School of Public Health, Boston, MA, USA

^12^School of Medicine, University of Missouri-Kansas City, Kansas City, MO, USA

1. **Introduction**

In the main paper, we discuss image-based technologies, such as multiplex immunofluorescence (mIF), becoming crucial for assaying the TIME and allowing for multiple markers to be assessed in a single experiment. Despite the advancements in technology, there are challenges that arise in the data generated from these images. To address these challenges, we developed Bayesian hierarchical multi-cell type analysis models that incorporate the relationships between the cell types while modeling them simultaneously. These models were applied to three large epidemiologic cohorts: Nurses’ Health Study I and II (NHSI/II), African American Cancer Epidemiology Study (AACES), and the University of Colorado Ovarian Cancer Study (UCOCS). The main paper evaluated the results from the NHSI/II analysis and the AACES and UCOCS analyses are assessed here in the supplemental material.

1. **Prior Distributions**

The priors for the Bayesian multi-cell type model were carefully selected following extensive sensitivity analyses to ensure they were weakly informative. This approach provides a balance between model flexibility and avoiding the pitfalls of overly diffuse or restrictive priors. Importantly, the use of weakly informative priors enhances the model’s generalizability across diverse cancer types, making it adaptable to a range of the TIME. Additionally, these tailored priors contributed to improved computational efficiency, facilitating faster convergence during model fitting. These priors were also utilized for the respective counterparts in the single-cell type model.

The prior distribution for $\gamma_{i}$ in $p_{igj}\sim Beta\left( \gamma_{i}\pi_{ig},\gamma_{i}\left( 1-\pi_{ig} \right) \right)$ follows $\gamma_{i}\sim Gamma(7,0.1)$. The prior distributions on the means of the $\beta_{0}$ and $\beta_{1}$ distributions are $v_{0}\sim MVN\left( \mu_{0},\delta_{0}I_{m} \right)$ and $v_{1}\sim MVN\left( \mu_{1},\delta_{1}I_{m} \right)$, where $\mu_{0}\sim MVN(logit\left( \varphi\right),I_{m})$, $\mu_{1}\sim MVN(0,I_{m})$, $\varphi$ is a user dependent value between 0 and 1 (subjective value for cell type proportions), $\delta_{0}\sim InvGamma(2,0.2)$, $\delta_{1}\sim InvGamma(2,0.2)$, and $I_{m}$ is the $m\times m$ identity matrix. The $\varphi$ value chosen for this work is 0.01 since immune cell activity for ovarian cancer is low with average cell population proportions being ~0.01. For the unstructured model, the covariances follow $T_{0}\sim InvWish(m+1,I_{m})$ and $T_{1}\sim InvWish(m+1,I_{m})$. For the scaled tree model, the $\lambda$ scalar follows $\lambda\sim Gamma(0.5,0.1)$. In the multi-level tree model, the prior distribution for $\omega_{l}$ follows $\omega_{l}\sim Gamma(0.5,0.1)$.

1. **Preliminary Results**

**Supplemental Figure 1** presents the differences in the expected log predictive density (ELPD) between the highest scoring model and all other models as applied to AACES, like **Figure 2** in the main paper. Similar to the results for the NHSI/II preliminary analysis, the over-dispersed models (BB, NB, ZIBB, ZINB) had smaller ELPD differences indicating a better performance in terms of model fit. A similar finding was observed in the analysis of UCOCS when comparing the 8 distributional models (**Supplemental Figure 2**). This further supports the use of the beta-binomial distribution as the base distribution for the Bayesian hierarchical multi-cell type analysis model.

No compelling evidence that stage of cancer was strongly associated with the abundance level of any of the immune cell types based on the best fitting models (BB, NB, ZINB and ZIBB). However, models which are commonly used for this type of analysis but fit our two datasets poorly (Binomial, Poisson) indicated potentially spurious but statistically significant association of abundance of several immune cell types with cancer stage. As an illustrative example, **Supplemental** **Figure 3** presents the results for macrophages (marker CD68) from UCOCS. As with the other cell types in the UCOCS analysis, the 95% credible intervals (CI) contain zero. As expected, the CIs for the over-dispersed distributions are wider than the models that don’t explicitly model the over-dispersion. This marker shows the impact of using a poorly fitting model that does not account for the over-dispersion in the data. As presented, the poorly fitting models without accounting for overdispersion have the narrowest CI and lead to incorrect conclusions that the marker abundance is associated with stage of the cancer.

1. **Simulation Study**

**Table S1** displays the results for adjusting the distances in the covariance structure for the exponential decay, tree, scaled tree, and multi-level tree models, comparing the original distances to doubling those distances (0.5 🡪 1 and 1 🡪 2). The moderate perturbation level was utilized for this component of the simulation study. Overall, doubling the distances did not significantly change the results. The results in **Table S1** show that the model comparison between the different set of distances had little to no effect on the significance proportions, ELPD estimates, and the CI ranges.

1. **Comparison of single-cell type analysis to multi-cell type analysis**

Like **Figure 1** in the main paper, **Supplemental Figure 4 & 5** exhibits a flowchart of the multi-cell modeling process using the immune cell types of interest from the AACES and UCOCS studies, respectively.

The results from the AACES analysis with stage of cancer, age at diagnosis, and debulking status as the predictors are shown in **Supplemental Figures 6-8**. Unlike the NHSI/II analysis, there was no block design included in the AACES analysis, leaving us with the single-cell model and the five separate multi-cell models. In **Supplemental Figure 6**, we see that there is no significance detected between stage of cancer and the immune cell population for the three cell types: T-cells, cytotoxic T-cells, and T-regs. The first column in **Supplemental Figures 7 & 8** also exhibit no significant association between the age at diagnosis and debulking status, respectively, and the immune cell populations. We also analyzed the width of the CIs, similar to the NHSI/II analysis, for each predictor as shown in plots in the second column of **Supplemental Figures 6-8**. The plots in these figures show that at least one of the multi-cell models had the smallest width of the CIs.

The results from the UCOCS analysis with stage of cancer, age at diagnosis, and debulking status as the predictors are shown in **Supplemental Figures 9-11**. Like the AACES analysis, there was no block design included in the UCOCS analysis leaving six models in the analysis. In plots in the first column of **Supplemental Figures 9-11**, we see that there is no significance detected between the predictors (stage, age, and debulking status, respectively) and the immune cell population for the four cell types (T-cells, cytotoxic T-cells, B-cells, and macrophages) of interest from the UCOCS data. Evaluating the width of the CIs in the plots in the second column of **Supplemental Figures 9-11** for each predictor shows higher accuracy with the multi-cell models compared to the single-cell model.

1. **How to use BICAM function within *BTIME***

The *BICAM* function in the *BTIME* R package is the function developed for implementing the Bayesian beta-binomial multi-cell type analysis model developed in this manuscript and is publicly available on the Comprehensive R Archive Network (CRAN) and through the Fridley Lab GitHub page. The main inputs for this function include the dataset (with the subject IDs, total cell count, predictor variable, and the cell abundances, in that order), the number of parameters/markers of interest, number of adaptation iterations for initializing the JAGS model, number of burn-in iterations, and the number of posterior sampling iterations. Once the function’s run is complete, the output will be a list of 6 items that are the posterior samples for each monitored parameter of the model, the time it took to complete the sampling, the exact JAGS model string, the initial values for each parameter, the dataset input into the function, and the monitored parameters. On the GitHub page is a tutorial on how to set up the observed data to properly input into the model, an example of what the outputs of the function look like, and details what each of the covariance structures are and how to appropriately construct these structures to input into the function.

**References**

1. Couzin-Frankel J. *Breakthrough of the year 2013. Cancer immunotherapy*. Science. 2013. 342(6165):1432-1433.
2. Ribas A, Wolchok JD. *Cancer immunotherapy using checkpoint blockade*. Science. 2018. 359(6382):1350-1355.
3. Aliazis K, Christofides A, Shah R, et al. *The tumor microenvironment’s role in the response to immune checkpoint blockade*. Nat Cancer. 2025. 6(6):924-937.
4. Jaillon S, and Di Mitri D. Editorial: *Profiling the tumour microenvironment to unveil biomarkers and develop novel therapeutics for cancer therapy*. Front. Med. 2023. 10:1178532.
5. Parra ER, Uraoka N, Jiang M, et al. *Validation of multiplex immunofluorescence panels using multispectral microscopy for immune-profiling of formalin-fixed and paraffin-embedded human tumor tissues*. Scientific reports. 2017. 7(1):13380.
6. Gorris MAJ, Halilovic A, Rabold K, Van Duffelen A, et al. *Eight-Color Multiplex Immunohistochemistry for Simultaneous Detection of Multiple Immune Checkpoint Molecules within the Tumor Microenvironment*. J Immunol. 2018. 200(1):347-354.
7. Schildkraut JM, Alberg AJ, Bandera EV, et al*. A multi-center population-based case-control study of ovarian cancer in African-American women: the African American Cancer Epidemiology Study (AACES)*. BMC cancer. 2014. 14:688.
8. Jordan KR, Sikora MJ, Slansky JE, et al. *The Capacity of the Ovarian Cancer Tumor Microenvironment to Integrate Inflammation Signaling Conveys a Shorter Disease-free Interval*. Clin Cancer Res. 2020. 26(23):6362-6373.
9. Steinhart B, Jordan KR, Bapat J, et al. *The Spatial Context of Tumor-Infiltrating Immune Cells Associates with Improved Ovarian Cancer Survival.* Molecular cancer research MCR. 2021. 19(12):1973-1979.
10. Wrobel J, Ghosh T. *VectraPolarisData: Vectra Polaris and Vectra 3 multiplex single-cell imaging data*. R package version 1.0.0. 2022.
11. Belanger CF, Hennekens CH, Rosner B, et al. *The nurses' health study*. Am J Nurs 78. 1978. 78(6):1039-1040.
12. Birmann BM, Barnard ME, Bertrand KA, et al. *Nurses' Health Study Contributions on the Epidemiology of Less Common Cancers: Endometrial, Ovarian, Pancreatic, and Hematologic*. American journal of public health. 2016. 106(9):1608-1615.
13. Boutot ME, Purdue-Smithe A, Whitcomb BW, et al. *Dietary Protein Intake and Early Menopause in the Nurses' Health Study II*. Am J Epidemiol. 2018. 187(2):270-277.
14. Hecht JL, Kotsopoulos J, Hankinson SE, et al. *Relationship between epidemiologic risk factors and hormone receptor expression in ovarian cancer: results from the Nurses' Health Study*. Cancer Epidemiol Biomarkers Prev. 2009. 18(5):1624-1630.
15. Hathaway CA, Conejo-Garcia JR, Fridley BL, et al. *Measurement of Ovarian Tumor Immune Profiles by Multiplex Immunohistochemistry: Implications for Epidemiologic Studies*. Cancer Epidemiol Biomarkers Prev. 2023. 1;32(6):848-853.
16. Bürkner PC. *brms: An R Package for Bayesian Multilevel Models Using Stan*. Journal of Statistical Software. 2017. 80(1):1–28.
17. Stan Development Team. *RStan: The R interface to Stan* (R package version 2.32.7). 2025. https://mc-stan.org/
18. Vehtari A, Gelman A, Gabry J. *Practical Bayesian model evaluation using leave-one-out cross-validation and WAIC*. Statistics and Computing. 2017. 27(5):1413-1432.
19. Gelman, A., Carlin, J. B., Stern, H. S., & Rubin, D. B. *Bayesian Data Analysis* (2nd ed.). Chapman & Hall/CRC. 2004.
20. Bender, M. A., Farach-Colton, M., Pemmasani, G., Skiena, S., & Sumazin, P. *Lowest Common Ancestors in Trees and Directed Acyclic Graphs*. Journal of Algorithms. 2005.
21. Denwood MJ. *runjags: An R Package Providing Interface Utilities, Model Templates, Parallel Computing Methods and Additional Distributions for MCMC Models in JAGS*. Journal of Statistical Software. 2016. 71(9):1–25.
22. Drake CG, Lipson EJ, Brahmer JR. *Breathing new life into immunotherapy: review of melanoma, lung and kidney cancer*. Nat Rev Clin Oncol. 2014. 11(1):24-37.
23. Menon S, Shin S, Dy G. *Advances in Cancer Immunotherapy in Solid Tumors.* Cancers (Basel). 2016. 8(12):106.
24. Emens LA, Ascierto PA, Darcy PK, et al. *Cancer immunotherapy: Opportunities and challenges in the rapidly evolving clinical landscape*. Eur J Cancer. 2017. 81:116-129.
25. Havel JJ, Chowell D, Chan TA. *The evolving landscape of biomarkers for checkpoint inhibitor immunotherapy*. Nat Rev Cancer. 2019. 19(3):133-150.
26. Dobosz P, Stępień M, Golke A, et al. *Challenges of the Immunotherapy: Perspectives and Limitations of the Immune Checkpoint Inhibitor Treatment*. International Journal of Molecular Sciences. 2022. 23(5):2847.
27. Tao W, Sun Q, Xu B, et al. *Towards the Prediction of Responses to Cancer Immunotherapy: A Multi-Omics Review*. Life. 2025. 15(2):283.
28. Luo R, Chyr J, Wen J, et al*.* *A novel integrated approach to predicting cancer immunotherapy efficacy*. Oncogene. 2023. 42(23):1913-1925.
29. Zhang H, Hunter MV, Chou J, et al. *BayesTME: An end-to-end method for multiscale spatial transcriptional profiling of the tissue microenvironment*. Cell Syst. 2023 Jul 19. 14(7):605-619.
30. Najera-Zuloaga J, Lee DJ, Arostegui I. *A beta-binomial mixed-effects model approach for analysing longitudinal discrete and bounded outcomes*. Biom J. 2019 May. 61(3):600-615.
31. Wrona M V, Ghosh R, Coll K, et al. *The 3 I’s of immunity and aging: immunosenescence, inflammaging, and immune resilience*. Frontiers in Aging. 2024. 5.
32. Liu Y, Yu Y, Chen Y, et al. *Tumor immunosenescence driven by chronic inflammation: Mechanisms, microenvironment remodeling and therapeutic strategies*. Aging and Disease. 2025.
33. Kim NH, Sim SJ, Han HG, et al. *Immunosenescence and age-related immune cells: causes of age-related diseases*. Archives of Pharmacal Research. 2025. 48:132–149.

| **Table S1:** Summary of the significance proportions, ELPD, and CI ranges for the exponential decay, tree, scaled tree, and multi-level tree models with the original distances (shown in **Figure 2** of the main manuscript) and double the original distances for each cell-type using the moderate perturbation level (20%) from the 100 simulations. The models were compared to themselves just with different distances from the immune differentiation path with the model that performed better highlighted in green. The yellow boxes indicate that both models had equal results. | | | | | | | | | | | | |
| --- | --- | --- | --- | --- | --- | --- | --- | --- | --- | --- | --- | --- |
|  | **Proportion of Significance** | | | | **# of Sim. with highest ELPD** | | | | **# of Sim. with Narrowest CI** | | | |
| **Model** | **M1** | **M2** | **M3** | **M4** | **M1** | **M2** | **M3** | **M4** | **M1** | **M2** | **M3** | **M4** |
| Exp. Decay | 0.11 | 0.09 | 0.07 | 0.06 | 44 | 43 | 67 | 63 | 53 | 51 | 46 | 63 |
| Exp. Decay  (2x distance) | 0.09 | 0.08 | 0.08 | 0.04 | 56 | 57 | 33 | 37 | 47 | 49 | 54 | 37 |
| Tree | 0.13 | 0.08 | 0.08 | 0.06 | 51 | 39 | 36 | 57 | 53 | 48 | 44 | 57 |
| Tree  (2x distance) | 0.11 | 0.07 | 0.09 | 0.07 | 49 | 61 | 64 | 43 | 47 | 52 | 56 | 43 |
| Scaled Tree | 0.12 | 0.08 | 0.06 | 0.05 | 46 | 53 | 54 | 48 | 46 | 62 | 53 | 75 |
| Scaled Tree  (2x distance) | 0.10 | 0.08 | 0.06 | 0.04 | 54 | 47 | 46 | 52 | 54 | 38 | 47 | 25 |
| Multi-Level Tree | 0.12 | 0.08 | 0.08 | 0.07 | 50 | 44 | 62 | 62 | 55 | 50 | 61 | 44 |
| Multi-Level Tree  (2x distance) | 0.11 | 0.06 | 0.09 | 0.07 | 50 | 56 | 38 | 38 | 45 | 50 | 39 | 56 |

**Supplemental Figure Legends:**

**Supplemental Figure 1:** The difference in ELPD measurements from the best fitting model for the 8 models fit to the various immune markers assessed in the AACES ovarian cancer study. Binomial (B), Poisson (P), Beta-Binomial (BB), Negative Binomial (NB), Zero-inflated Binomial (ZIB), Zero-inflated Poisson (ZIP), Zero-inflated Negative Binomial (ZINB), and zero-inflated Beta-Binomial (ZIBB). Overall, the best fitting models were models where over-dispersion was explicitly modeled (ZIBB, ZINB, BB and NB).

**Supplemental Figure 2:** The difference in ELPD measurements from the best fitting model for the 8 models fit to the various immune markers assessed in the Colorado ovarian cancer study. Binomial (B), Poisson (P), Beta-Binomial (BB), Negative Binomial (NB), Zero-inflated Binomial (ZIB), Zero-inflated Poisson (ZIP), Zero-inflated Negative Binomial (ZINB), and zero-inflated Beta-Binomial (ZIBB). Overall, the best fitting models were models where over-dispersion was explicitly modeled (ZIBB, ZINB, BB and NB).

**Supplemental Figure 3:** 95% credible intervals for macrophages cell type (marker CD68) association with stage (Early vs. Late) in the Colorado ovarian cancer study. The intervals are much narrower for the models that do not account for over-dispersion (B, P, ZIB, ZIP) leading to an incorrect conclusion that the level of macrophages in the tumor is associated with stage of the cancer.

**Supplemental Figure 4:** Illustration of the modeling framework for the Bayesian multi-cell type model using the protein markers from the AACES ovarian cancer study. The hierarchical cell type tree or immune cell differentiation pathway (left) shows the relationship between the cell populations. The abundance of all cell populations is modeled simultaneously in the Bayesian multi-cell type model incorporating the relationship between cell populations.

**Supplemental Figure 5:** Illustration of the modeling framework for the Bayesian multi-cell type model using the protein markers from the UCOCS ovarian cancer study. The hierarchical cell type tree or immune cell differentiation pathway (left) shows the relationship between the cell populations. The gray cell icons (Hematopoietic Stem Cells [HSC], Common Myeloid Progenitor [CMP], and Cyclophilin-Related Cell Proteins [CCP]) are not included in the analysis as they are utilized to depict the immune differentiation paths between the cell types of interest. The abundance of all cell populations is modeled simultaneously in the Bayesian multi-cell type model incorporating the relationship between cell populations.

**Supplemental Figure 6:** The 95% credible intervals for the $\beta_{1}$ estimates (first column) and the widths of those intervals (second column) for each cell type from the AACES analysis for the single-cell type analysis model (“Single-cell”) and the multi-cell type analysis models with stage of cancer (Early vs. Late) as the predictor. For the multi-cell type models, “Unstr” is the unstructured model, “Exp” is the exponential decay model, “Tree” is the tree model, “TreeScaled” is the scaled tree model, and “TreeMultiLevel” is the multi-level tree model. The red dashed lines for the plots in the first column indicate the *x*-intercept at zero. Intervals that do not contain zero indicate a significance between predictor (stage) and immune cell infiltration. In the second column, the red bar indicates the model with the smallest width.

**Supplemental Figure 7:** The 95% credible intervals for the $\beta_{1}$ estimates (first column) and the widths of those intervals (second column) for each cell type from the AACES analysis for the single-cell type analysis model (“Single-cell”) and the multi-cell type analysis models with age at diagnosis as the predictor. For the multi-cell type models, “Unstr” is the unstructured model, “Exp” is the exponential decay model, “Tree” is the tree model, “TreeScaled” is the scaled tree model, and “TreeMultiLevel” is the multi-level tree model. The red dashed lines for the plots in the first column indicate the *x*-intercept at zero. Intervals that do not contain zero indicate a significance between predictor (age) and immune cell infiltration. In the second column, the red bar indicates the model with the smallest width.

**Supplemental Figure 8:** The 95% credible intervals for the $\beta_{1}$ estimates (first column) and the widths of those intervals (second column) for each cell type from the AACES analysis for the single-cell type analysis model (“Single-cell”) and the multi-cell type analysis models with debulking status (Suboptimal vs. Optimal) as the predictor. For the multi-cell type models, “Unstr” is the unstructured model, “Exp” is the exponential decay model, “Tree” is the tree model, “TreeScaled” is the scaled tree model, and “TreeMultiLevel” is the multi-level tree model. The red dashed lines for the plots in the first column indicate the *x*-intercept at zero. Intervals that do not contain zero indicate a significance between predictor (debulking status) and immune cell infiltration. In the second column, the red bar indicates the model with the smallest width.

**Supplemental Figure 9:** The 95% credible intervals for the $\beta_{1}$ estimates (first column) and the widths of those intervals (second column) for each cell type from the UCOCS analysis for the single-cell type analysis model (“Single-cell”) and the multi-cell type analysis models with stage of cancer (Early vs. Late) as the predictor. For the multi-cell type models, “Unstr” is the unstructured model, “Exp” is the exponential decay model, “Tree” is the tree model, “TreeScaled” is the scaled tree model, and “TreeMultiLevel” is the multi-level tree model. The red dashed lines for the plots in the first column indicate the *x*-intercept at zero. Intervals that do not contain zero indicate a significance between predictor (stage) and immune cell infiltration. In the second column, the red bar indicates the model with the smallest width.

**Supplemental Figure 10:** The 95% credible intervals for the $\beta_{1}$ estimates (first column) and the widths of those intervals (second column) for each cell type from the UCOCS analysis for the single-cell type analysis model (“Single-cell”) and the multi-cell type analysis models with age at diagnosis as the predictor. For the multi-cell type models, “Unstr” is the unstructured model, “Exp” is the exponential decay model, “Tree” is the tree model, “TreeScaled” is the scaled tree model, and “TreeMultiLevel” is the multi-level tree model. The red dashed lines for the plots in the first column indicate the *x*-intercept at zero. Intervals that do not contain zero indicate a significance between predictor (age) and immune cell infiltration. In the second column, the red bar indicates the model with the smallest width.

**Supplemental Figure 11:** The 95% credible intervals for the $\beta_{1}$ estimates (first column) and the widths of those intervals (second column) for each cell type from the UCOCS analysis for the single-cell type analysis model (“Single-cell”) and the multi-cell type analysis models with debulking status (Suboptimal vs. Optimal) as the predictor. For the multi-cell type models, “Unstr” is the unstructured model, “Exp” is the exponential decay model, “Tree” is the tree model, “TreeScaled” is the scaled tree model, and “TreeMultiLevel” is the multi-level tree model. The red dashed lines for the plots in the first column indicate the *x*-intercept at zero. Intervals that do not contain zero indicate a significance between predictor (debulking status) and immune cell infiltration. In the second column, the red bar indicates the model with the smallest width.

**Supplemental Figure 1:**

**
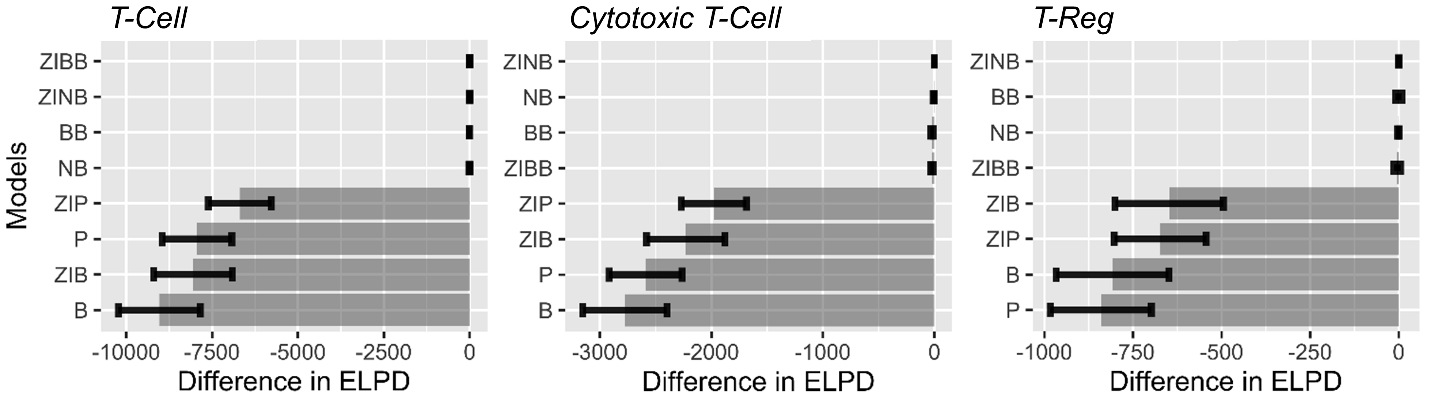
**

**Supplemental Figure 2:**

**
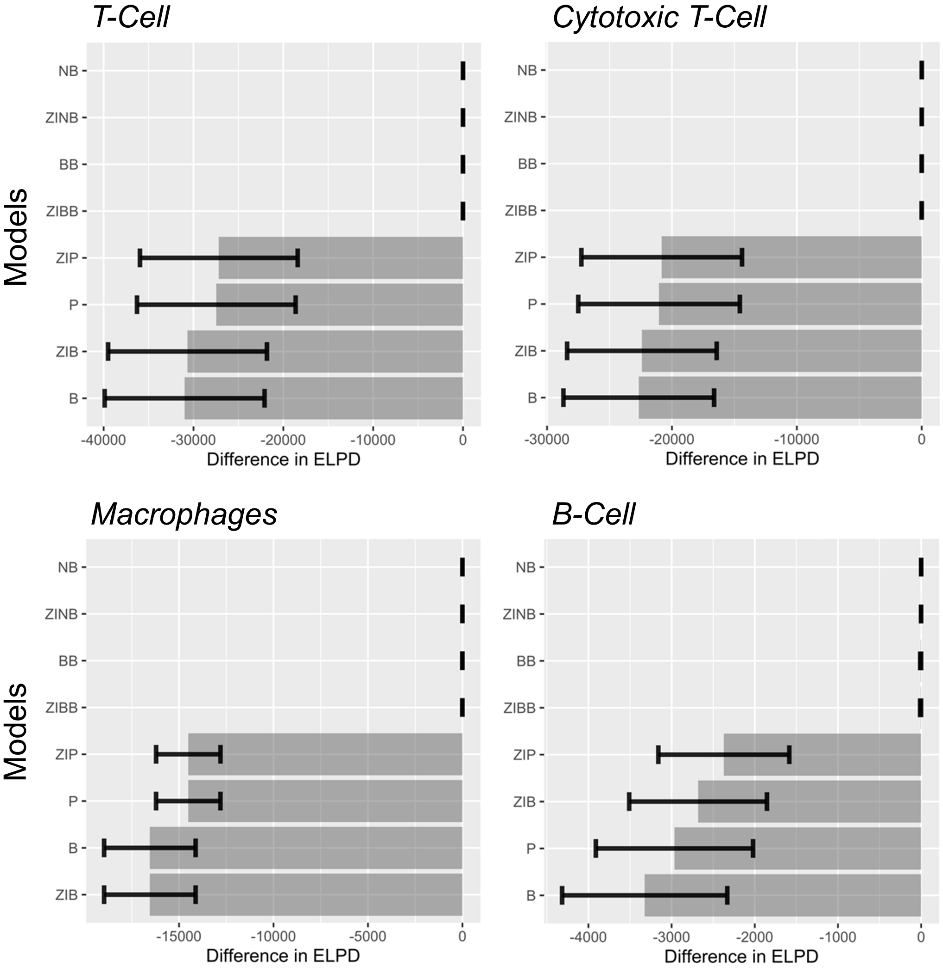
**

**Supplemental Figure 3:**


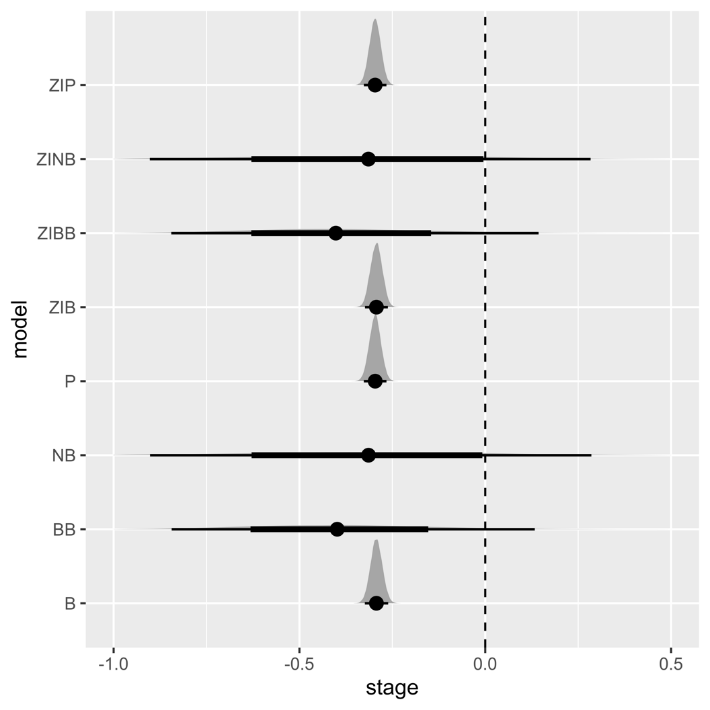


**Supplemental Figure 4:**

**
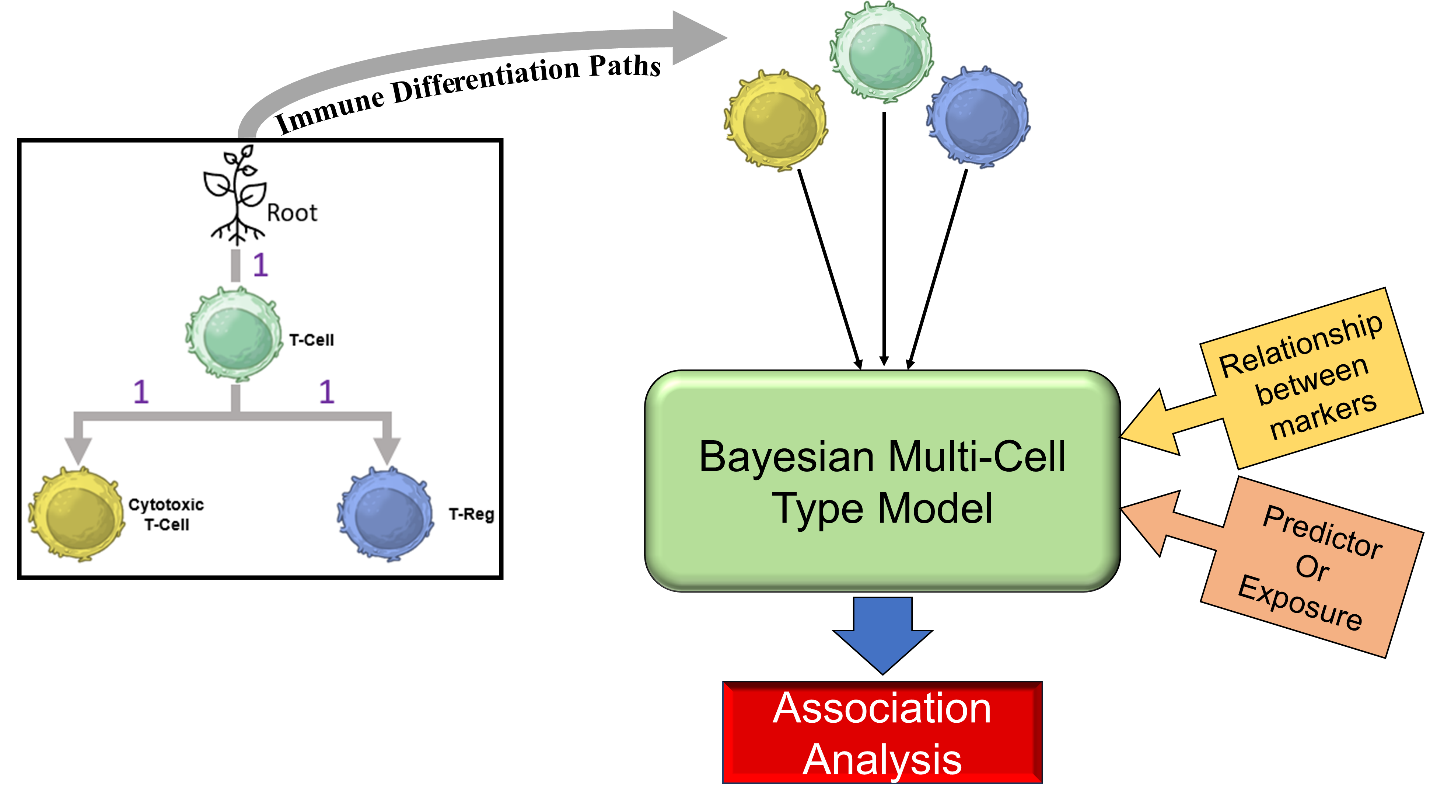
**

**Supplemental Figure 5:**

**
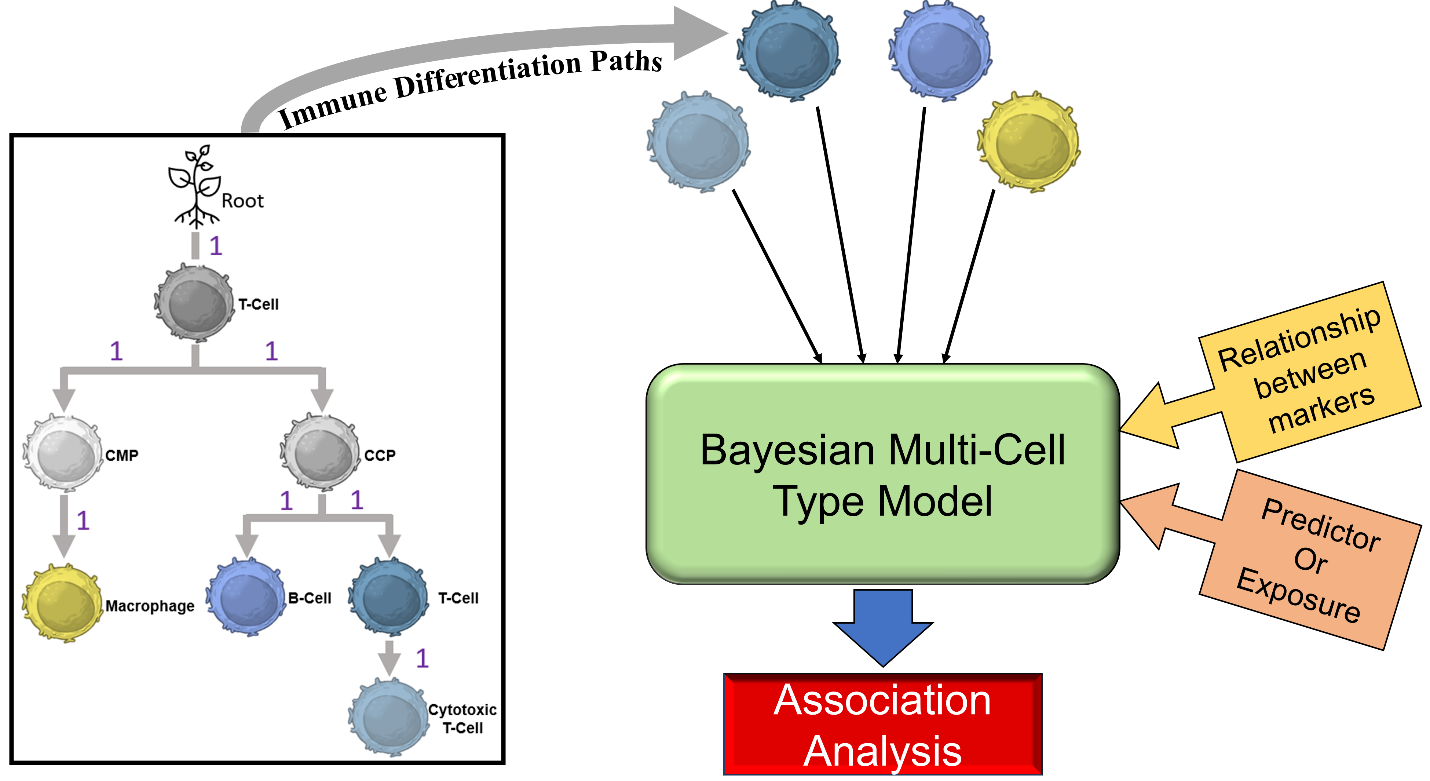
**

**Supplemental Figure 6:**

**
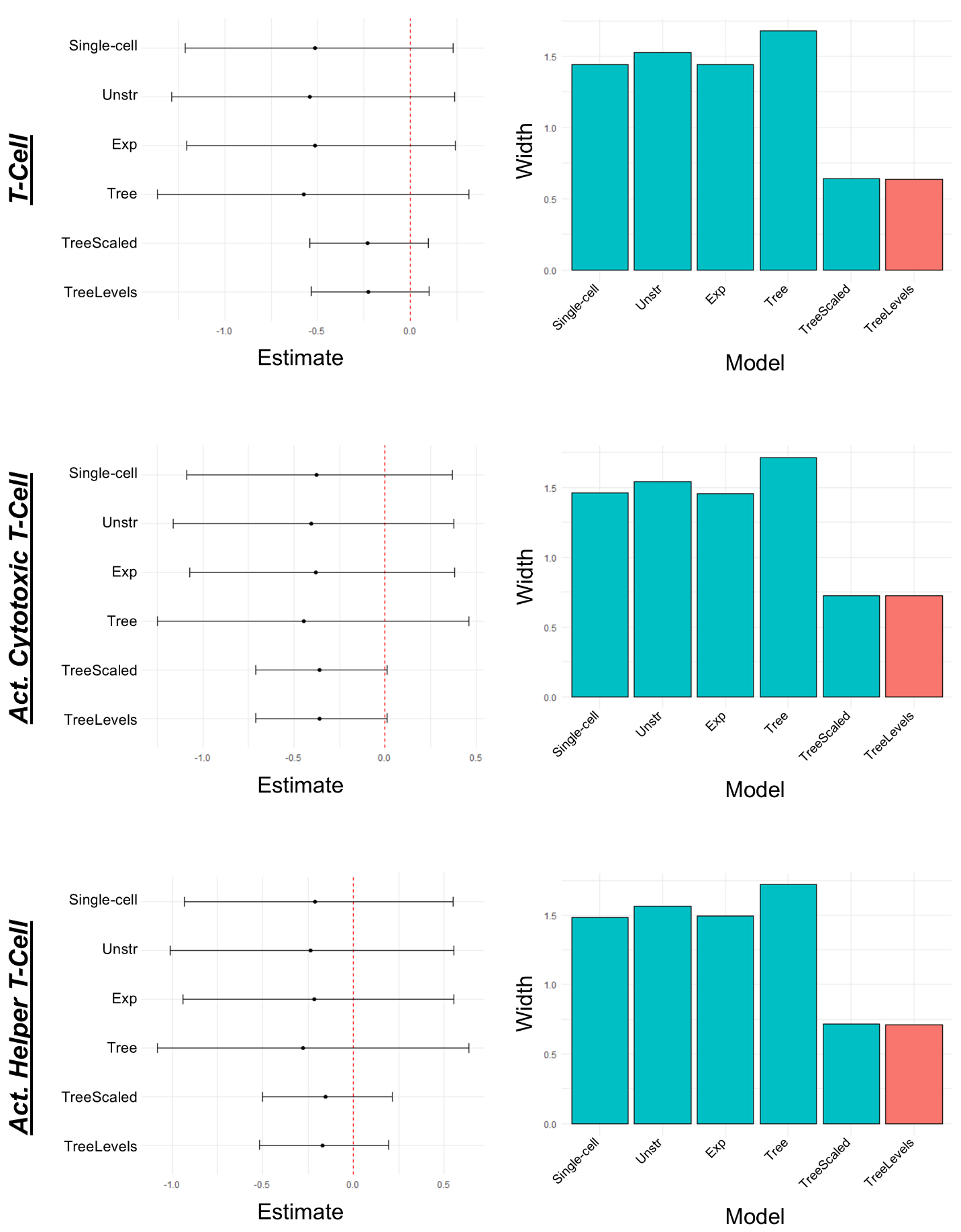
**

**Supplemental Figure 7:**

**
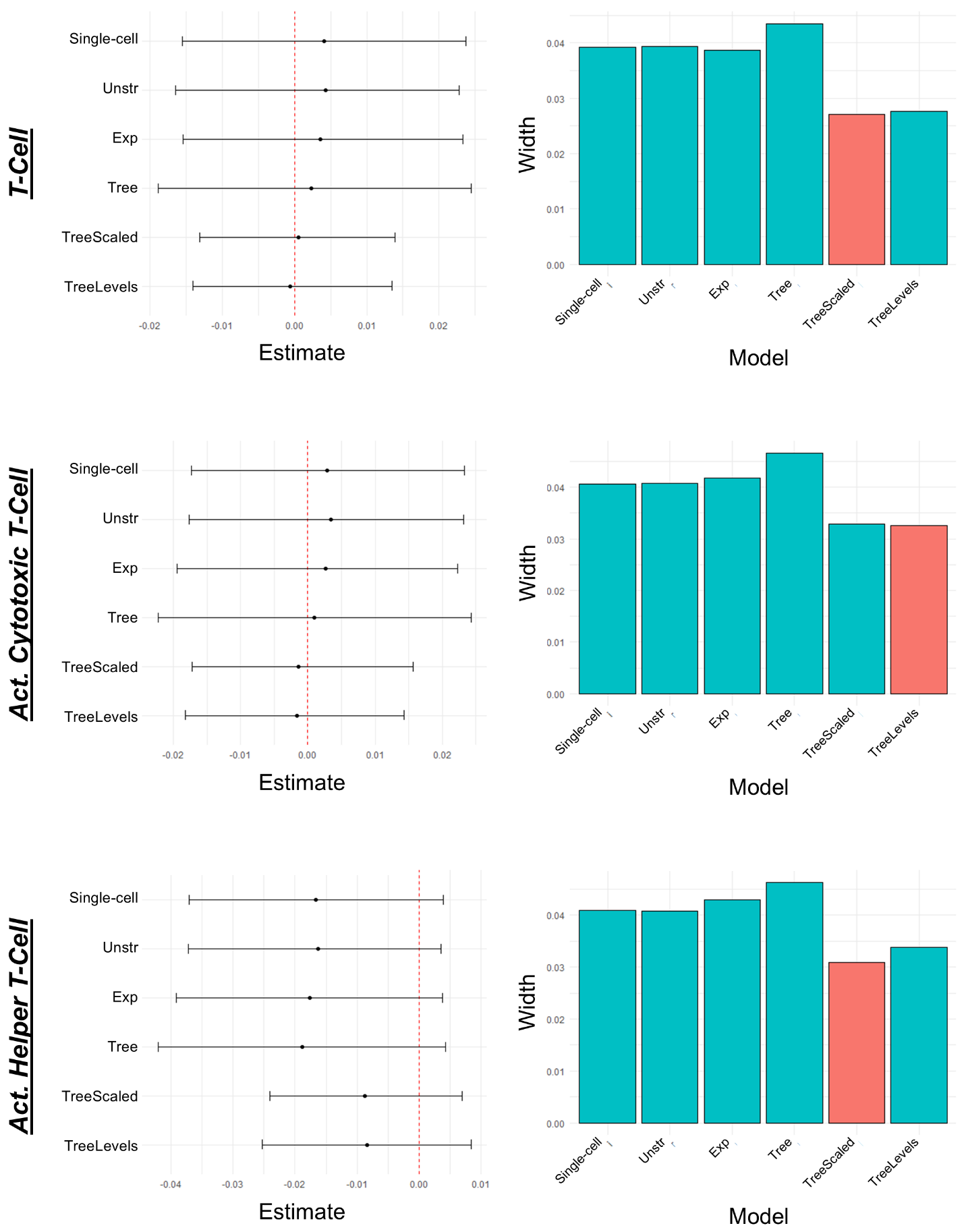
**

**Supplemental Figure 8:**


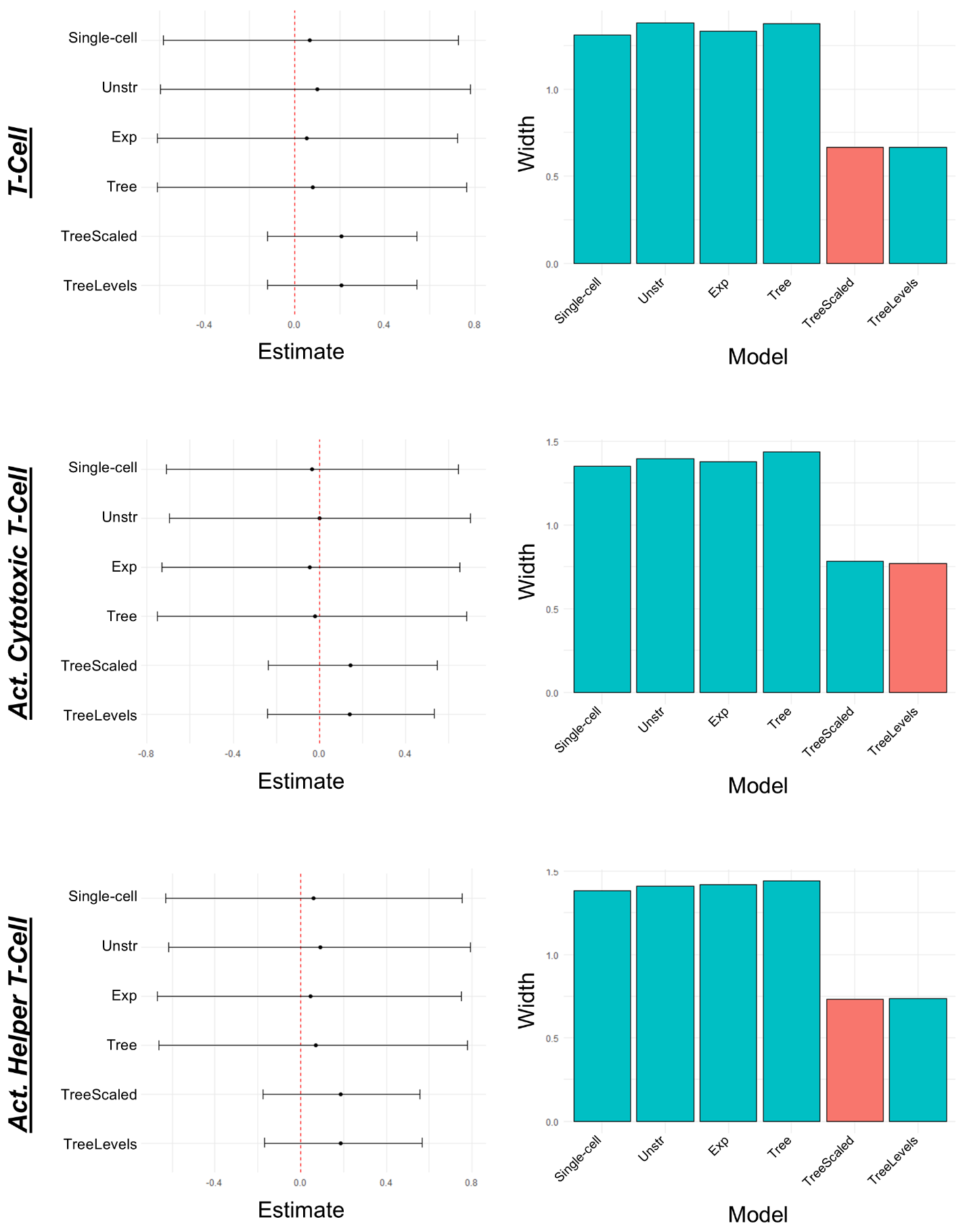


**Supplemental Figure 9:**


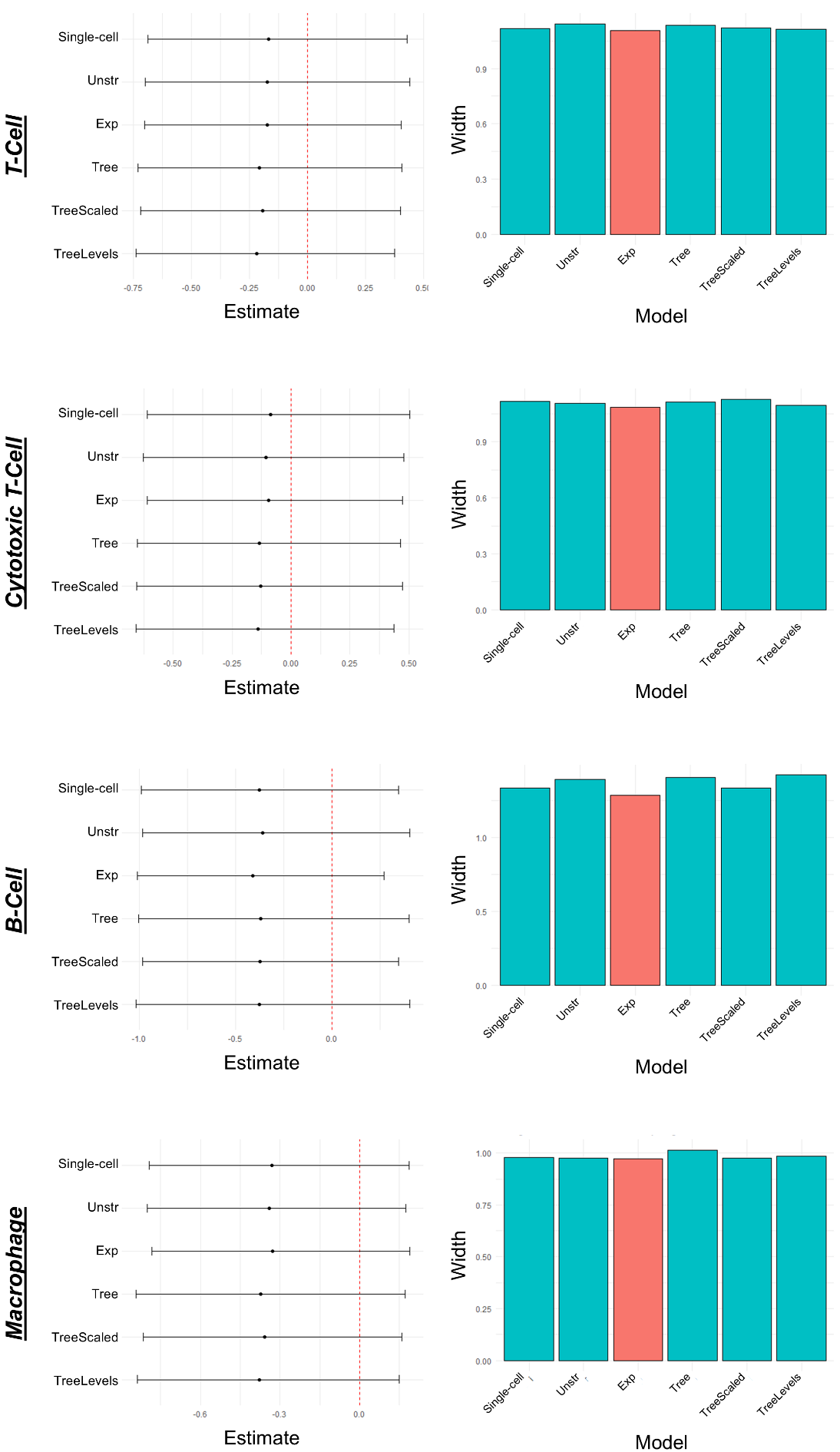


**Supplemental Figure 10:**


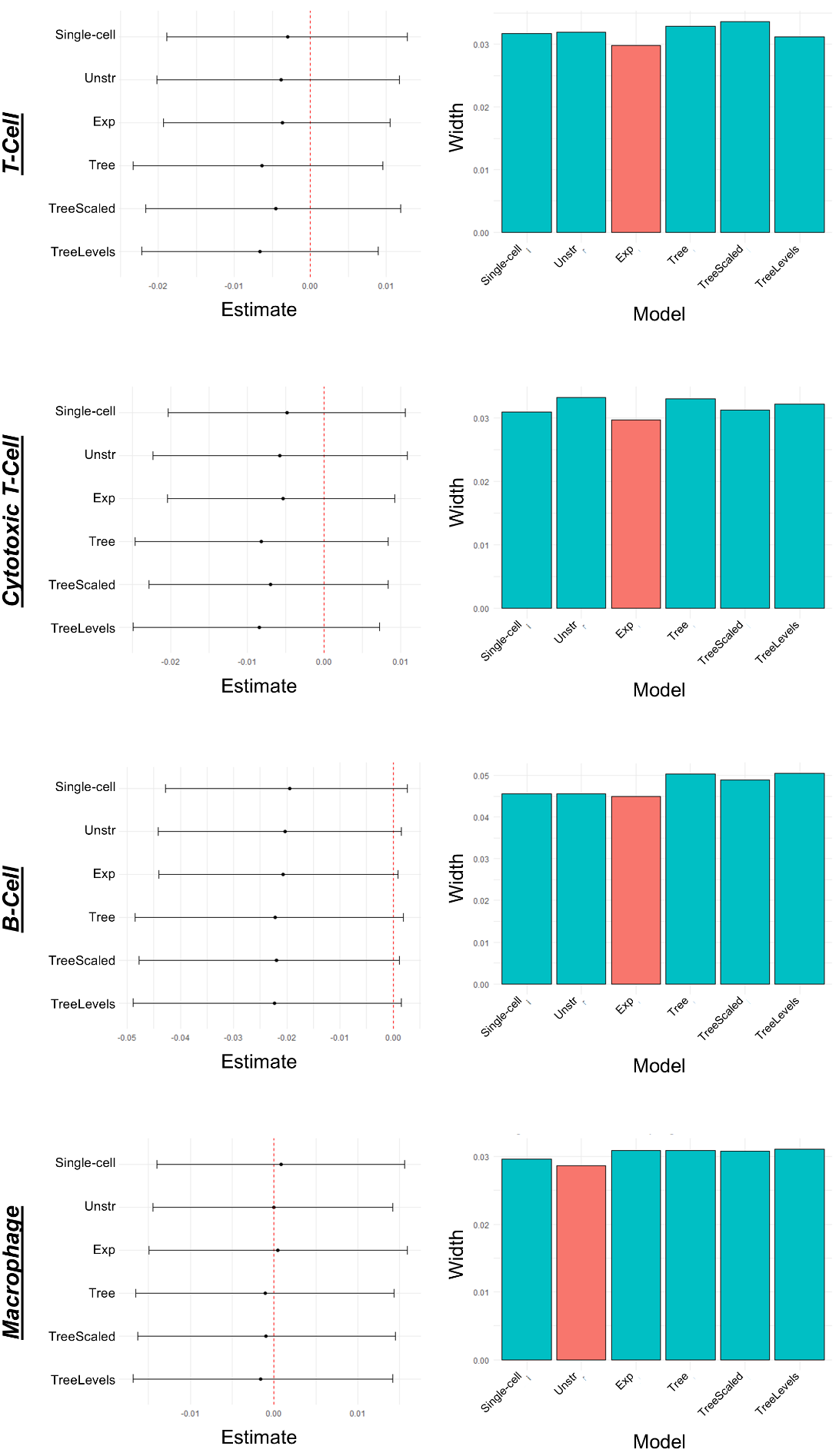


**Supplemental Figure 11:**


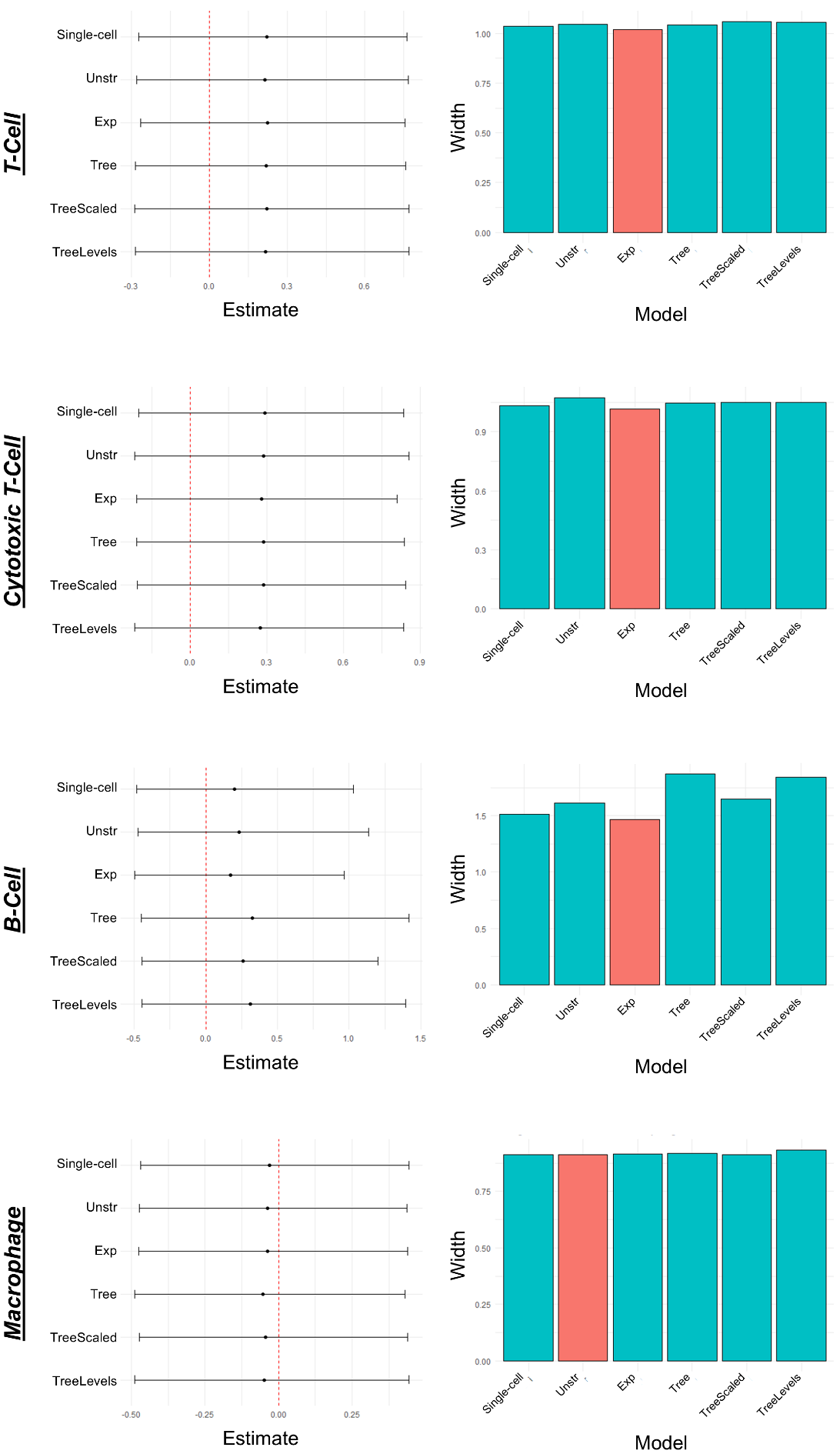

Supplement: BayesBB_Manuscript_Supplement_Final_bbag053 [file bayesbb_manuscript_supplement_final_bbag053.docx]
